# PAIRWISE: Deep Learning-based Prediction of Effective Personalized Drug Combinations in Cancer

**DOI:** 10.21203/rs.3.rs-8518203/v1

**Published:** 2026-01-19

**Authors:** Chengqi Xu, Ilkay Us, Jake Cohen-Setton, Marta Milo, Ben Sidders, Jude Fitzgibbon, Ari M. Melnick, Heng Pan, Krishna C. Bulusu, Olivier Elemento

**Affiliations:** 1. Englander Institute for Precision Medicine, Weill Cornell Medicine, New York, NY, USA; 2. Meinig School of Biomedical Engineering, Cornell University, Ithaca, NY, USA; 3. The Affiliated Stomatological Hospital of Nanjing Medical University, Nanjing, China; 4. Oncology Data Science, AstraZeneca, Cambridge, UK; 5. Hematology R&D, AstraZeneca, Waltham, MA, USA; 6. Division of Hematology and Oncology, Department of Medicine and Meyer Cancer Center, Weill Cornell Medicine, New York, NY, USA; 7. State Key Laboratory of Female Fertility Promotion, Center for Reproductive Medicine, Department of Obstetrics and Gynecology, Peking University Third Hospital, Beijing, China; 8. Turbine AI, Budapest, Hungary

## Abstract

Combination therapies offer promise for improving cancer treatment efficacy and preventing recurrence. Preclinical screening strategies can prioritize synergistic drug combinations. However, identifying optimal drug combinations tailored to specific cancer subtypes and individual patients is extremely challenging due to the vast number of possible combinations and tumor heterogeneity. To address this gap, we combined deep learning with transfer learning to incorporate prior scientific knowledge and predicted drug synergy based on tumor-specific transcriptome profiles. PAIRWISE explicitly modeled synergistic effects of drug combinations in cancer cell lines or individual tumor samples based on drug chemical structures, drug targets, and transcriptomes of inferred samples. Of note, PAIRWISE outperformed competing models with an AUROC (the area under the receiver operating characteristic curve) of 0.847 on held-out cancer cell lines. Moreover, when applied to an independent dataset of combinations with Bruton Tyrosine Kinase inhibitors (BTKi) in Diffuse Large B Cell Lymphoma (DLBCL) cell lines, PAIRWISE accurately predicted synergistic drug combinations with an AUROC of 0.720. To further confirm the robustness of PAIRWISE predictions, we selected 30 approved or investigational agents for DLBCL treatment and validated their synergy with BTKi across eight non-Hodgkin lymphoma cell lines. The synergistic predictions of PAIRWISE showed strong concordance with *in vitro* screening results. These findings highlight PAIRWISE’s potential as a powerful in silico tool to prioritize candidate personalized drug combinations for further experimental validation.

## Introduction

Combination therapies using multiple anti-cancer drugs together have become a cornerstone of cancer treatment^1^. Regimens combining chemotherapy, targeted agents, and immunotherapies have improved cancer outcomes in the last decades^2–5^. However, identifying optimal drug combinations for each cancer type and individual patient remains challenging. The search space of possible combinations is vast, and responses can vary greatly due to tumor heterogeneity. High-throughput screening (HTS) provides an unbiased approach to testing large numbers of combinations, but usually lacks patient-derived cell models and cannot identify patient-specific combinations^2,6–9^. Current empirical methods for designing combinations also have limitations in predicting patient-specific synergistic regimens. There is thus an unmet need for accurately predicting personalized synergistic drug combinations.

Advanced computational methods could greatly reduce the search space and minimize the experimental efforts required to determine effective drug combinations^10^ .Traditional machine learning (ML) models could learn functional mappings between high-dimensional input data and drug synergy measured by the Loewe or Bliss additivity model^10^, requiring extensive feature selection before training^11–14^. Recent deep learning (DL) models have adopted a multimodal architecture^15–30^, bypassing the reliance on single data modalities. However, extant models rarely use pre-trained techniques to represent inherent features of both chemical and sample-specific transcriptional modalities. It is challenging for them to accurately predict drug synergy in cancers with limited cases in the training set.

To address these gaps, we developed a DL model PAIRWISE, which employed a multimodal architecture to integrate different modalities influencing drug responses, including chemical structures, drug targets, and transcriptomes of individual tumor samples. Modalities were encoded with specialized neural networks and integrated using attention mechanisms that focus learning on relevant features. PAIRWISE also utilized transfer learning, wherein the model is pre-trained on large drug and transcriptome datasets, thus learning generalized representations of drug chemical structures and sample-specific transcriptomes.

We first applied PAIRWISE and benchmarked methods to a curated drug combination screening dataset collected from 13 public sources across hematological malignancies and solid tumors^31^. The curated dataset was named the p13 dataset and has ~200,000 screened drug combinations across 167 cell lines. Compared with benchmarked methods, PAIRWISE achieved better performance across several metrics including AUROC (the area under the receiver operating characteristic curve) and AUPRC (the area under the precision-recall curve) when drugs, drug combinations, or cancer cell lines were held out. Moreover, PAIRWISE showed high sensitivity and specificity for predicting synergistic drug combinations containing Bruton Tyrosine Kinase inhibitors (BTKi) in an independent dataset^32^. Here, we experimentally demonstrated that several of the novel synergistic BTKi combinations are indeed synergistic in Diffuse Large B Cell Lymphoma (DLBCL) cell lines. Combinations of BTKi with agents targeting DNA Damage Response (DDR) pathways demonstrated marked synergy in aggressive subtypes of DLBCL. Finally, PAIRWISE was applied to transcriptome profiles of primary patient DLBCL tumors (LS cohort) and predicted candidate drugs with synergy with BTKi, such as PARP and DNA-PK inhibitors. These predictions suggest potential therapeutic strategies targeting DNA damage response (DDR) pathways in aggressive DLBCL subtypes, providing valuable insights for personalized treatment options^33^ PAIRWISE represents a novel DL framework for identifying optimal drug combinations tailored to specific cancer subtypes and individual patients, paving the way toward an enhanced understanding of the role of personalized drug combinations in cancer.

## Results

### PAIRWISE is a novel DL model for personalized drug combination prediction

To predict patient-specific effective drug combinations, we developed PAIRWISE - an integrative DL approach that considers both the mechanism of drug combination and transcriptome of individual cancer samples. PAIRWISE can predict whether two compounds or drugs are likely to achieve synergistic cell killing in individual cancer samples, even for drugs that are not present in the combination screening data. To fulfill this objective, PAIRWISE uses transfer learning to first ground the model in general domain knowledge before fine-tuning it on our curated large-scale combination screening data. Also, a multimodal architecture allows PAIRWISE to learn higher-order relationships among drug chemical structures, drug target interactions (DTIs), and sample-specific transcriptome.

**Fig. 1:**
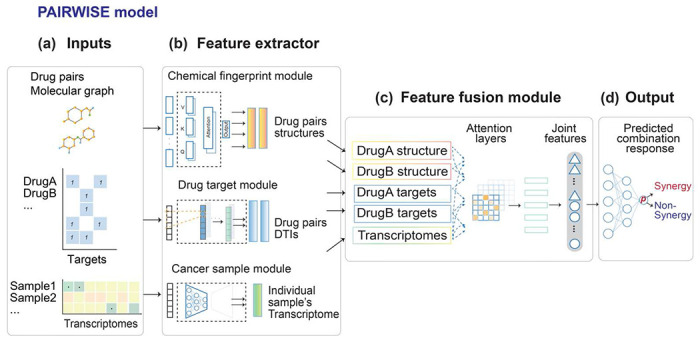
The PAIRWISE model for predicting drug synergy. **(a)** PAIRWISE takes three inputs: the drug combination molecular graphs representing chemical information as adjacency matrices, which capture the atoms and chemical bonds of the input compounds structures. The drug target is generated as an equallength vector recording the binary status whether that input drug has a specific drug target. The cancer sample used transcriptome values as input, which denote the individualized transcriptome profile of that sample. **(b)** In feature extractor module, for the chemical fingerprint branch, a chemical foundation model is trained on 10 millions of molecule structures, and it is fine-tuned to guide the drug pairs structures optimization; for the drug target branch **(c)** In the feature fusion module, two vectors representing drug structures embeddings, two vectors representing drug targets embedding, and a single vector representing cancer sample with the same length are aligned and updated in the attention layer. The updated embeddings from an attention mechanism are linearized into a joint feature embedding vector. **(d)** In the output layer, the join feature embedding is passed into a multi-layer perceptron to produce the predicted synergy probabilities, and consequently measure a given drug combination effect between synergy and non-synergy status of an individualized cancer sample.

Specifically, PAIRWISE takes three sets of features as input: a combination of two drug chemical structures, known protein targets for each compound (Drug Target Interactions, or DTIs), and the transcriptome profile of an individual sample (Fig. 1a). PAIRWISE outputs the binary prediction of the combination effect (synergistic or not) of two drugs in the inferred sample. To create vectorized input features, we represented drug chemical structures using the Simplified Molecular Input Line Entry System (SMILES)^34,35^. SMILES were then converted into molecular graphs encoded as adjacency matrices, in which each node represents an atom and each edge represents a chemical bond between atoms. DTIs were first converted into binary vectors recording whether a gene is a direct or indirect target (1 indicates yes while 0 indicates no) across all known targets of at least one drug (*n* = 4,098) in Drug Target Commons (DTCs)^36^. Recognizing the limitations of viewing targets in isolation, we employed network propagation using the STRING v11 protein-protein interaction (PPI) network. This process transformed the binary data into a continuous interaction matrix (2,614 drugs x 4,098 targets), providing a richer, network-level perspective that captures how drugs might influence interconnected biological pathways beyond their direct targets. Transcriptome profiles of individual samples were represented as an expression matrix of 4,079 genes (see Methods). Vectorized features were then fed into embedding layers of PAIRWISE. Embedding layers serve as feature extractors and consist of three modules: (1) the chemical fingerprint module, (2) the DTI module, and (3) the cancer sample module (Fig. 1b). The chemical fingerprint module leverages transfer learning techniques built upon a chemical pre-trained foundation model (PFM)^37^. This general model was pre-trained on 11 million chemical compounds, which can enable reasonable parameter initiation and easy fine-tuning. Compared with simple chemical descriptors such as SMILES or Morgan fingerprints^38^, chemical PFMs provide more sophisticated representations of atoms and chemical bonds. Parameters of the chemical PFM were updated and used to guide PAIRWISE after fine-tuning with the p13 dataset. The parameters update was frozen in the test dataset. The DTI module was implemented with a convolutional neural network (CNN), which condensed 4,098 drug targets into 500 features that contain critical information about DTI. We chose 500 dimensions after systematically evaluating various embedding sizes (**see** Methods). In the cancer sample module, PAIRWISE represented transcriptomes of individual samples using an auto-encoder pre-trained on *in vitro* cell-line samples from the Cancer Cell Line Encyclopedia (CCLE) ^39^ and patient samples from The Cancer Genome Atlas (TCGA) ^40^ with corresponding transcriptomes profiles. Instead of the raw expression matrix, the pre-trained autoencoder learns shared biological signals between datasets common to cell-line and patient data. In this way, the knowledge learned from the training samples can be transferred to a new sample. Next, an attention-based feature fusion module was used to combine features in each branch (Fig. 1c). In the attention layer, each feature embedding was applied with a scaled dot product-based attention with the embedding of other modalities to produce an update feature embedding. The five resulting embeddings were combined across all three branches in a single-layer neuron to produce joint features, which were then fed into an output layer to generate the binary prediction of synergy for a particular drug combination in a given sample (drug combinations with a probability > 0.5 were classified as synergistic) (Fig. 1d).

### PAIRWISE outperforms state-of-the-art models across cancer cell lines from different tissues

To optimize the parameters of PAIRWISE, we first aggregated 13 public HTS assays of drug combinations using unique criteria (**see** Methods; Supplementary Table. 1). Synonyms of each compound were determined by the Tanimoto similarity of Morgan fingerprints and were assigned with unique DrugBank IDs (Supplementary Fig. 1). We determined using Morgan fingerprints since target similarities are quite low (Supplementary Fig. 2). Cell lines and synonyms were assigned unique DepMap IDs using a namemapping table derived from the DepMap portal^39^ (Supplementary Fig. 1). Synergy scores of drug combinations were calculated based on dose-response metrics, including Bliss, HSA, Loewe, and ZIP. This curated dataset (p13) contained 279,452 unique drug combinations, 2,614 drugs, and 167 cancer cell lines. We also implemented an end-to-end pipeline to assist in the development and evaluation of drug combination prediction models (Supplementary Fig. 2).

**Fig. 2:**
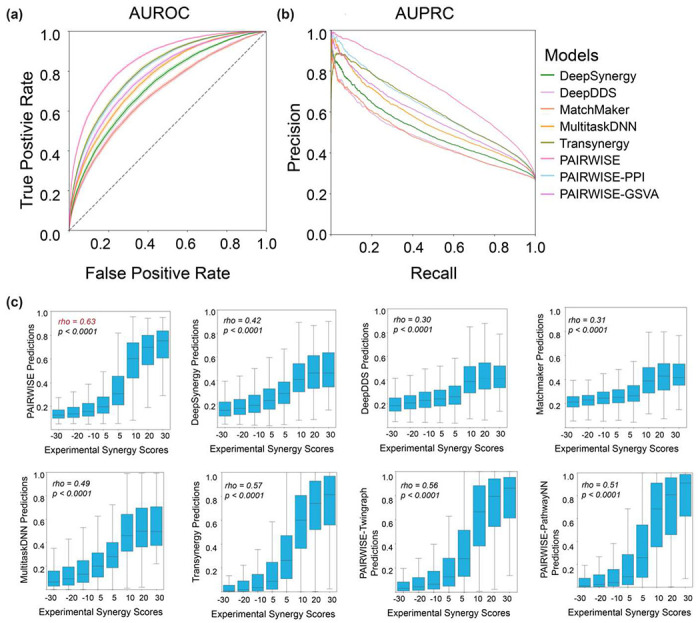
Predictive performance. **(a-b)** The performance of the suite of predictive models for drug synergy across metrics. For each model, which predicts drug synergy as defined by each respective model, the performance was measured by AUROC and AUPRC. **(c)** Predicted versus actual drug combination synergy scores across all (cell line, drug combinations) tuples studied. Box plots show each bin’s 25th, 50th, and 75th percentiles; whiskers show maximum and minimum values.

To evaluate the performance of PAIRWISE, we tested our model through 3 distinct settings and benchmarked five published DL models (Supplementary Table. 2; Supplementary Fig. 3). In each setting, the p13 dataset was equally split into 5 folds, which do not share common drug combinations (leave-combo-out), drugs (leave-drug-out), or cancer samples (leave-sample-out) (Table 1). Specifically, we employ a 5-fold cross-validation within each setting, where the training set is further divided into training and validation subsets using a 90/10 ratio. These settings were used to evaluate the ability of models to generalize from the training data to unseen drug combinations, individual drugs, or cancer samples. In the leave-sample-out setting, 167 cell lines were divided into 4 clusters based on the expression of the top 1,000 most variable genes used an affinity propagation method. The biggest cluster with 93 cell lines was used as the training set, and the remaining cell lines were used as the test set. PAIRWISE showed superior performance compared to other models when assessed using AUROC and AUPRC in all three settings (Fig. 2a–b). Before being converted to binary classifications, PAIRWISE and benchmarked methods outputted continuous probabilities of drug synergy. PAIRWISE also achieved the highest Spearman correlation between predicted synergy probabilities and experimental synergy scores in all settings (Fig. 2c; Table 2).

To assess PAIRWISE performance in diverse tissues, we explored the performance of the model in 10 different cancer types of the p13 test dataset. The AUROC showed consistent robust performance (0.795 ~ 0.867) across all cancer types (Supplementary Fig. 4a). By contrast, we found that the published model (Multitask-DNN), which specifically designed for transfer learning in tissues with limited training data such as bone and prostate cell lines, has limited performance (Supplementary Table. 3). PAIRWISE predictions exhibited strong correlations (Spearman’s Coefficient, 0.508-0.606) with experimental synergy scores across different tissues (Supplementary Fig. 4b). We also found that PAIRWISE maintained consistently high AUROC performance across tumor types with variable amount of training data, including in cancers with limited training data (Supplementary Fig. 5). We conclude that PAIRWISE learns cross-tissue generalizable patterns of drug combinations, and provides accurate prediction of combination treatments.

### PAIRWISE benefits from the module representation methods for drug synergy prediction

To systematically evaluate the importance of each of the three input and representation modules in PAIRWISE, we replaced individual modules with alternatives. Several PAIRWISE variant models were created, and their predictive performance was compared to PAIRWISE on the p13 dataset. For the chemical fingerprint branch, we replace the Chemical PFM with SMILES (PAIRWISE-SMILES) or Morgan (PAIRWISE-Morgan) Fingerprints to represent a drug compound chemical structure. For the drug target module, we replaced DTCs with DrugBank^41^ (PAIRWISE-DrugBank), a widely used database, as well as an alternative approach to propagate DrugBank with PPI (PAIRWISE-PropagatedDrugbank). For the cancer sample module, we introduced two different cancer sample representations based on PPI and Gene set variation analysis (GSVA) scores as surrogate pre-training techniques (see Methods; Supplementary Fig. 6). Compared with all the variant models, PAIRWISE had the best synergy prediction accuracy on the p13 dataset (Table 3). Replacing pre-trained TCGA encoders with PPI and GSVA decreases the performance of synergy prediction. Of note, the AUPRC of PAIRWISE-SMILES and PAIRWISE-Morgan were the lowest among all the models, suggesting the chemical finger module featured with chemical PFM may have the most significant contribution to the prediction accuracy in PAIRWISE. To more directly isolate the contribution of chemical structure information, we created an ablated model called PAIRWISEabCF. This variant excludes the chemical fingerprint module entirely while retaining the drug target and cancer sample feature modules. This analysis allowed us to determine the effects of chemical structures on model performance. We trained and tested the ablated model on the p13 dataset under identical conditions. We observed that PAIRWISEabCF performed significantly worse than the full PAIRWISE across all metrics (Supplementary Table. 4). Further analysis indicated that this performance degradation was linked to poorer model training and generalization. Specifically, PAIRWISEabCF exhibited a higher training loss compared to the full PAIRWISE model (e.g, 0.3 vs. 0.2 at epoch 50), suggesting more erroneous predictions during training (Supplementary Fig. 7a,b). Moreover, a quantile-quantile analysis revealed that PAIRWISEabCF’s synergy predictions were skewed toward extreme values, indicative of overfitting and reduced generalizability when chemical structures are omitted (Supplementary Fig. 7c).

Finally, we developed and evaluated a PAIRWISE variant, termed PAIRWISE-Mut, which exclusively utilizes mutation data. This was meant to explore whether a gene expression based approach is superior to genomic-based approach in predicting drug synergy. PAIRWISE-Mut maintains the same overall architecture as the original expression-based PAIRWISE. For PAIRWISE-Mut, genomic mutation data were sourced from the Cancer Cell Line Encyclopedia (CCLE) and The Cancer Genome Atlas (TCGA) databases. These data were processed into a gene-level binary status (1 for mutated, 0 for wild-type) based on five types of nonsynonymous mutations: missense and nonsense mutations, frameshift insertions and deletions, and splice site alterations. In total, 1,083 frequently mutated genes were included as features. An autoencoder, integral to the PAIRWISE architecture, was then pre-trained specifically on this genomic mutation data before being integrated with drug chemical structures and drug target representations. PAIRWISE-Mut was subsequently trained on the p13 dataset using the same procedures as the original expression-based PAIRWISE model. A comparative performance analysis revealed that PAIRWISE-Mut performed comparably to the original expression-based PAIRWISE model when evaluated on metrics such as AUROC, and AUPRC (Supplementary Fig. 8a). Specifically, PAIRWISE-Mut achieved an AUROC of 0.853, which was only slightly lower than the 0.855 AUROC achieved by the expression-based PAIRWISE model. Furthermore, as the training set size was incrementally increased, the performance of both these single-omic models (mutation-based and expression-based) showed similar positive trends (Supplementary Fig. 8b). We subsequently investigated whether an ensemble model combining predictions from both PAIRWISE-Mut and the expression-based PAIRWISE could offer enhanced performance. The resulting ensemble model, integrating both mutation and expression data, demonstrated only a marginal improvement in predictive performance compared to each single-omics model evaluated independently (Supplementary Fig. 8c). These findings indicate that while genomic mutation data can yield a model (PAIRWISE-Mut) with strong predictive capabilities comparable to an expression-based model, the latter (PAIRWISE) may offer slightly better performance. Furthermore, the combination of both data types in an ensemble showed limited additive benefit in our framework.

### Clinically Tested Combinations Receive Higher PAIRWISE Scores

We hypothesized that if PAIRWISE genuinely captures biologically meaningful drug synergy, drug pairs that have already entered clinical trials and shown early signs of clinical benefit should, on average, receive higher predicted synergy scores than pairs never evaluated together in patients. To test this, we curated 196 drug combinations (downloaded on May 1, 2025) from the Drug Combination DataBase (DCDB version 2.0) and mapped each trial indication to a TCGA cancer type (**See** Methods; Supplementary Fig. 9a). After predicting synergy probabilities for every pair in every TCGA tumor and averaging within cancer types, we found that clinically validated combinations were consistently assigned higher scores than a background set of randomly selected, untested pairs: Kolmogorov–Smirnov statistics ranged from 0.114 in prostate cancer to 0.298 in renal cancer, with *p* < 0.001 in almost every tissue examined (Supplementary Fig. 9b). This strong, tissue-wide enrichment of high PAIRWISE scores among regimens already under clinical investigation in patients provides compelling external evidence that the model captures real-world drug synergy and therefore has genuine translational potential. The model’s ability to predict outcomes with *in vivo* relevance was confirmed in an independent patient-derived xenograft (PDX) dataset (Gao et al., 2015), where PAIRWISE-predicted synergy scores showed significant positive correlation with tumor response (r = 0.388, P < 0.001) and were associated with improved progression-free survival (log-rank test, *P* = 0.006) (Supplementary Text). These findings provide evidence that PAIRWISE predictions translate beyond in vitro contexts to more clinically relevant in vivo models.

### PAIRWISE accurately predicts drug synergy in an independent DLBCL HTS dataset

**Fig. 3:**
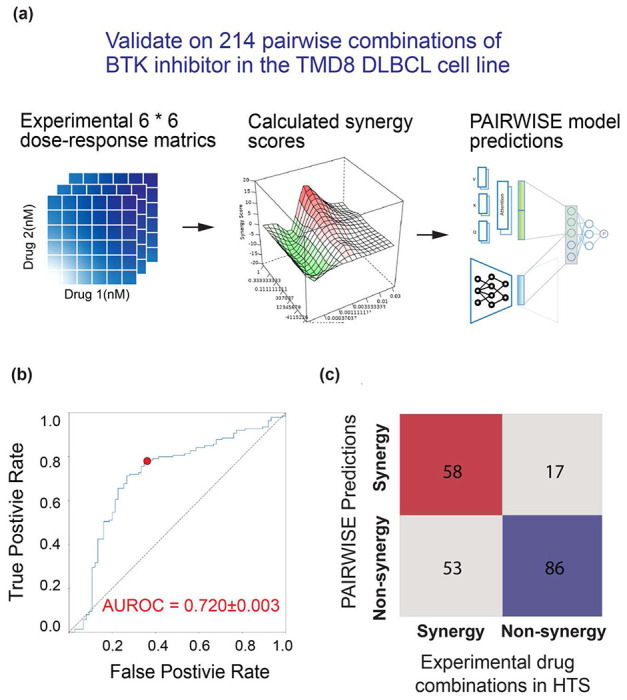
PAIRWISE predicts accurately combinatorial responses with the external experimental DLBCL HTS design. **(a)** Flowchart of the analysis procedure. **(b)** AUROC of PAIRWISE performance in distinguishing effective from ineffective drug combinations. (**c)** Confusion matrix for point indicated in (b) demonstrating the best performance of PAIRWISE against the DLBCL HTS dataset.

Next, we sought to assess the performance of PAIRWISE in an independent drug combination dataset. We used an HTS dataset testing the efficacy and synergy of a BTK inhibitor in DLBCL^32^ (Fig. 3a). The HTS study analyzed 466 combinations of ibrutinib with approved or investigational drugs in the TMD8 DLBCL cell line. After excluding compounds that lacked either an annotated chemical structure or any annotated gene target, 214 drugs were left for validation. In this dataset, PAIRWISE achieved a robust prediction accuracy of synergistic pairs (AUROC = 0.720±0.003, Fig. 3b–c). Of note, this task is very challenging since there were only a small number of records from 3 hematopoietic cell lines in the training cohort (*n* = 2,445 out of 279,452; 1% of the training cohort), and no records were from TMD8. Combinations that were predicted to have synergy showed significantly higher synergy in the experimental combinatorial viability assay, compared with those that were predicted to be only non-synergistic (*P* = 0.0014, Wilcoxon rank sum test). These results suggest that the model is still effective with cell lines or drug compounds that were never seen before by the model.

### High-throughput screening validates synergistic BTKi combinations recommended by PAIRWISE in DLBCL

**Fig. 4:**
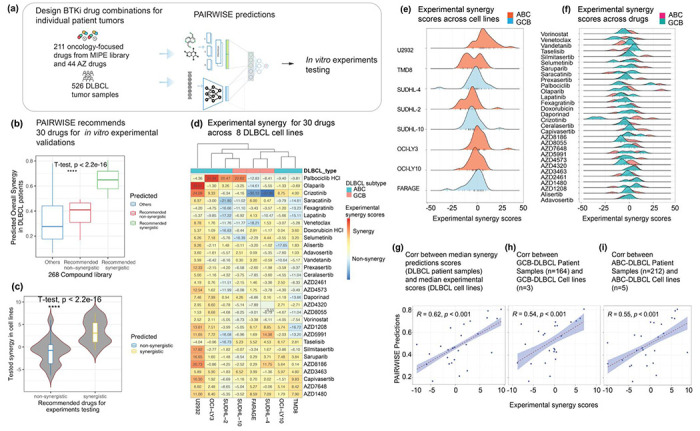
Experimental validation of top predicted combinations **(a)** Flowchart of the analysis procedure. **(b)** PAIRWISE analysis of 255 combinations between BTKi and drugs from MIPE library and AZ compounds identifies 30 drugs with predicted synergy **(c)** Experimental validation, quantified by Loewe synergy scores for predicted synergistic and predicted non-synergistic combinations. **(d)** Heatmap of experimental synergy scores for the predicted synergistic drug combinations, assessed in cell lines (of both ABC and GCB syubtypes). **(e)** Distribution of combination scores of each tested drug combination. (f) Experimental synergy scores for each combination, separating ABC and GCB subtypes. (**g-i**) Concordance between median synergy probability predictions in DLBCL patients and median measured synergy scores in DLBCL cell lines across recommended drug

One of the strengths of PAIRWISE is that despite being trained on cell lines, it can in theory be applied to primary patient samples. Encouraged by our results in DLBCL, we thus sought to predict drug combination synergy in 562 primary DLBCL patient samples where transcriptome profiles were available^33^. Most patients presented relatively high-risk disease: 45% with Ann Arbor stage III or IV, and 70% with IPI group intermediate or high. We continued to focus on combinations involving BTKi since there is continued interest in using them in DLBCL. PAIRWISE was applied to predict combination synergy of BTKi in conjunction with 211 drugs from NCATS MIPE library (a resource containing oncology-focused and clinic-prioritized targeted therapies) and 44 additional investigational drugs provided by Astra-Zeneca (Fig. 4a). In total, 30 out of 255 combinations were identified as recommendations for experiments based on the rank of average predicted synergy scores across all the samples (Fig. 4b). Predicted synergistic combinations included drugs that play critical roles in DNA repair, epigenetic modification, JAK/STAT signaling, and PI3K/AKT signaling.

We tested 30 predicted drug combinations in 8 DLBCL cell lines in 6 * 6 dose-response combination metrics to seek to confirm their synergy (Methods; Fig. 4d; Supplementary Table. 5). We found that synergistic drug combinations nominated by PAIRWISE were strongly and significantly enriched for synergistic cell killing outcomes, in contrast to combinations predicted to be non-synergistic combinations (Fig. 4c). As expected, the experimental synergies were heterogenous in different ABC and GCB DLBCL cell lines (Fig. 4e–f). Notably, we observed a high concordance between median predicted synergies in DLBCL patients and median experimental synergy scores in DLBCL cell lines across predicted drug combinations (Pearson’s Coefficient r=0.55, *P* < 0.001, Fig. 4g). This correlation was consistent in GCB and ABC DLBCLs (Pearson’s Coefficient r=0.54, *P* < 0.001, Fig. 4h; Pearson’s Coefficient r=0.62, *P* < 0.001, Fig. 4i). These observations confirm the ability of PAIRWISE to robustly predict drug synergy in different DLBCL subtypes. PAIRWISE identifies the combination of olaparib and saruparib, PARP inhibitors, with acalabrutinib as having more synergy in more aggressive ABC DLBCL. The capacity of PARP inhibitors to synergize with acalabrutinib, specifically by targeting and overcoming single drug resistances has recently been demonstrated for mantel cell lymphoma^42^. Interestingly, PAIRWISE also identifies a combination with acalabrutinib for which synergy is also associated with alterations in DDR pathway, such as the combination of DNA-PK inhibitor AZD7648. Recent studies established a connection between DDR activation and BCL-2 overexpression, serving as the biological underpinning for the unfavorable prognosis and chemoresistance evident in DLBCL subtypes^43^. PAIRWISE identifies a number of additional drugs that may be combined with acalabrutinib to the same effect, including the AKT inhibitor capivasertib, the multitargeted tyrosine kinase inhibitor crizotinib and cyclin-dependent kinase 4/6 inhibitor palbociclib.

**Fig 5:**
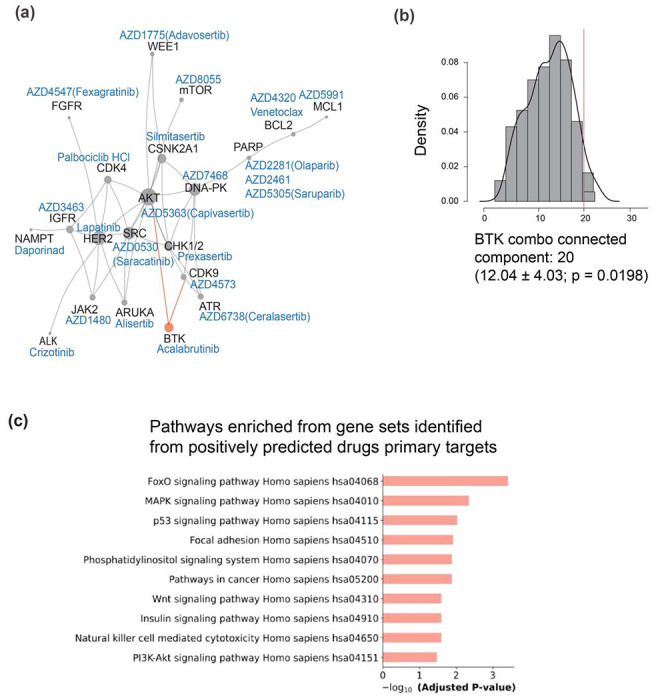
**(a)** Network of proteins targeted by our screening BTK combination drugs (highlighted by PAIRWISE). These proteins are not distributed randomly in the human interactome, but form a connected component and multiple subgraphs shown in the graph. **(b)** Distribution the connected component size for randomly selected groups of protein targets. The observed BTK combination component, whose size is indicated by the red arrow, is larger than expected by chance. **(c)** Pathway enrichment analysis of targets of BTKi synergy partners highlights distinct biological pathways significantly enriched (FDR-corrected p-value <0.05) in synergistic combinations, including signal transduction, apoptosis, and receptor-mediated signaling.

We then sought to search for molecular mechanisms associated with the experimentally validated BTKi drug combinations. We mapped the 29 candidate drug protein targets of the predicted positive BTKi combination drugs onto a human protein-protein interactome (PPI) assembled from 21 public databases. We found that 20 of the 29 involved target proteins form a large, connected component based on available PPIs (Fig. 5a). Using randomly selected sets of protein targets, we observed that the BTK interacting group is larger than expected by chance (*P* = 0.0198, Fig. 5b), indicating that targets of predicted BTKi synergy partners share mechanistic connections. We found that at least one of the predicted synergistic combination (BTKi+CHK1i - prexasertib) maps to a known synthetic lethal in the SynLethDB database (Supplementary Fig. 10). We then performed a comparative pathway enrichment analysis contrasting predicted synergistic vs. non-synergistic drug combinations with BTKi in DLBCL. We analyzed the top 30 synergistic combinations and 30 random non-synergistic controls. KEGG and GO enrichment analyses (FDR<0.05) revealed significant pathway enrichments only among synergistic combinations, notably FoxO, MAPK, p53, PI3K-Akt, insulin, Wnt signaling, focal adhesion, NK cell-mediated cytotoxicity, and cancer-associated phosphatidylinositol signaling pathways (Fig. 5c). No pathways were enriched among controls, underscoring mechanistic interactions among targets of predicted BTKi synergy partners.

### PAIRWISE-based combination stratification among patients with DLBCL

**Fig. 6:**
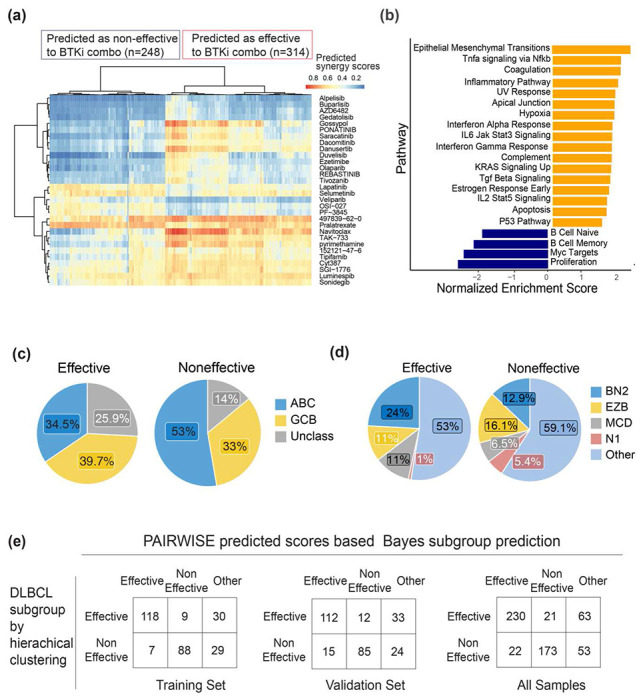
Identification and analysis of DLBCL patient subgroups with distinct predicted response to BTKi combination therapy. **(a)** The assignment of the DLBCL cases to the predicted effectiveness to BTKi combination based on the hierarchical clustering of predicted synergy scores by PAIRWISE. **(b)** Biological pathways most highly differentiated between DLBCL subgroups which are predicted to be effective or non-effective to BTKi combination. Pathways are sorted by their average ranking using GSEA analysis. **(c-d)** Distribution of predicted subgroups overlaps within gene-expression subgroups (ABC, GCB or other), or genetic subtypes (BH2, EZB, MCD, N1 or other). **(e)** The subgroup Bayes predictor are compared within the training, validation, and total set of samples.

Having validated the ability of PAIRWISE to make reliable predictions, we next sought to evaluate whether PAIRWISE could be used to stratify cancer patients into groups of patients responsive and non-responsive populations to BTKi drug combinations. Hierarchical clustering of predicted patient response (probability of synergy) to BTKi (acalabrutinib) combinations identified two patient subgroups, one with broad predicted response to BTKi combinations (BTKiCombo(+)) and one with limited predicted response (BTKiCombo(−)) (Fig. 6a). We found differences in survival upon R-CHOP between BTKiCombo(+) and BTKiCombo(−) tumors, suggesting biological differences between these two groups (Supplementary Fig. 11). To further characterize biological differences between the two groups, we sought to identify genes that were differentially expressed between BTKiCombo(+) and BTKiCombo(−) DLBCLs. Although attributions and trends for individual genes are informative, to gain further insights into regulatory pathways that are important to BTKi combination response, we applied pathway analysis (Methods) to determine pathways that are differentially represented between BTKiCombo(+) and BTKiCombo(−) DLBCL. Using Gene Set Enrichment Analysis (GSEA), we find pathways covering a broad spectrum of cellular processes and functions (Fig. 6b). Some of them are portions of well-known cancer pathways such as mTOR signaling, BCL2-regulated apoptosis, and epithelial-methylation transition (EMT). EMT has previously been linked to resistance to chemotherapy. When we correlated BTKiCombo(+) and BTKiCombo(−) with gene-expression subgroups (ABC, GCB or other), or genetic subtypes (BH2, EZB, MCD, N1 or other), we observed that non-BTKi combination responsive patients were enriched in ABC tumors and BTKi combination responsive patients were enriched in BN2 and MCD tumors (Fig. 6c–d). Since identifying whether a patient may respond to BTKi combinations may be valuable in the future, we sought to develop a linear predictor scores (LPS) algorithm for BTKiCombo-classification (see Methods). The final LPS algorithm incorporated 50 genes and correctly classified 0.733 of the training set tumors into the subgroup to which they had been assigned by hierarchical clustering. The reproducibility of the LPS algorithm was demonstrated by its ability to correctly classify 0.704 (95% CI = 0.611,0.786) of the tumors in the validation set (Fig. 6e, Supplementary Table. 6). Altogether, these results indicate that a molecularly distinct subtype of BTKi combination responsive DLBCL patients may exist and may be useful in guiding therapy choices in the clinic.

## Discussion

Machine learning has been used extensively to predict drug combinations based on the multiple omics data available for large panels of cancer cell lines, which are crucial to developing more robust preclinical biomarkers^11^. However, the translation of model predictions into clinical impact has been very limited owing to many factors including but not restricted to data-poor cancer types, lack of learning from clinical samples and imprecise predictions of drug combinations that have been encountered during model training. A wider, more integrative analysis, along with a model that is clinically predictive, is needed to understand drug combination response that delivers value to patients. In this study, we sought to address this issue with a new DL model, PAIRWISE, that enables individualized predictions on single cancer samples.

PAIRWISE consists of three modules, a chemical fingerprint module that takes as input molecular graphs, a drug target module that takes as input a binary vector of protein targets, and a cancer sample module that takes as input the transcriptome profile of an individual cell line and patient tumor. The key module fusion design of the model simulates drug combination inhibition on cells through an attention mechanism. This enables the model to generalize to novel data points and make accurate predictions based on chemical fingerprints and protein targets of drugs. PAIRWISE accurately captures drug combination responses *in vitro* and that translates to *in vivo* (for example, DLBCL patient tumors) and clinical settings. We evaluated PAIRWISE’s performance extensively on the gold standard combination data spanning over 200K drug combinations across 10 cancer types. PAIRWISE not only outperformed state-of-the-art models in the public domain but also has consistent performance across both data-rich and data-poor tissues, indicating that the model had learned generalizable and transferable knowledge from chemical structures, drug targets, and molecular profiles beyond the tissue of origin. One advantage of the current study is that our approach was applied to a dataset of drug-synergy measurements in a specific DLBCL cell population, indicating a precision medicine approach. Our *in vitro* experiments show that the extent of differentiation subgroups of DLBCL cells is an important factor for the prediction of the synergy that can be achieved with specific therapy combinations, which has potential clinical applications. The potential opportunities that could emerge from our approach could be exemplified through recent clinical studies highlighting the clinical translatability of PAIRWISE’s combination recommendations across hematological cancers– acalabrutinib plus AZD4573 (CDK9i) showed high response rates with a manageable safety profile in pretreated DLBCL patients in a dose escalation study^44^, acalabrutinib plus venetoclax (BCL2i) significantly improved progression-free survival in 1^st^ line Chronic Lymphocytic Leukemia patients^45^, and acalabrutinib plus ceralasertib (ATRi) in relapsed or refractory aggressive Non-Hodgkin’s Lymphoma in the PRISM study discontinued as of 2022 but a combination that has not yet been fully explored in DLBCL^46^. Previous study^33^ delineated distinctive DLBCL genetic subtypes with unique genotypic, epigenetic, and clinical attributes. The rise of Single-Cell RNA-Seq technology has illuminated DLBCL’s tumor heterogeneity. PAIRWISE predictions suggested for distinct subgroup DLBCLs with mutations involvement in the DNA damage response pathway, inhibitions to DDR and B Cell Receptor (BCR) pathways can be considered for potential therapeutic responses in DLBCL.

While our analyses indicate that PAIRWISE outperforms other approaches on the p13 curated dataset as well as on the Griner et al DLBCL HTDS dataset (AUROC is 0.720 for PARWISE and 0.610 for Transynergy, the next best model), PAIRWISE produces false positive predictions. Using cross-validation in the p13 dataset, PAIRWISE had on average a 5.6% False Positive Rate. When we applied PAIRWISE to the Griner et al DLBCL HTDS dataset, 17 drugs predicted to be synergistic by PAIRWISE, are nonsynergy by experimental HTS (a 23% False Positive Rate). Upon further inspection, we found that the majority (76 %) of the 17 false-positive predictions involved drugs not seen during training. By contrast, all the correctly classified compounds are actually in our training set. In addition, the Griner HTDS screen uses a DLBCL cell lines (TMD8) that is also not in PAIRWISE’s training set, and is transcriptionally distinct from the 182 cell lines in the training set (median pairwise Pearson’s Coefficient r=0.43 vs. 0.67 within-training set, P < 1 Ö 10-6, Mann-Whitney U). Taken together, these data indicate that despite good overall performance, as expected, out-of-distribution chemical and transcriptional space is a likely driver of misclassification in PAIRWISE. In the short term, predictions involving compounds or cell lines not in the training set should be treated with caution and systematically tested experimentally. In the long term, more data will likely be needed to train more robust models capable of improved generalization.

Future work could address some of the ’out-of-scope’ aspects and limitations of our study. For example, methods such as SHAP (SHapley Additive exPlanations) analysis could help identify which molecular features (specific genes, pathways, or chemical substructures) drive individual predictions. This may help identify mechanisms that are responsible for facilitating synergy. Future work should incorporate such explainability frameworks to generate mechanistic hypotheses alongside predictions. While we have shown that adding mutation data to transcriptome profile only marginally improves performances, there are additional levels of molecular information such as epigenetic states, metabolomic and proteomic profiles, or microenvironmental influences that could eventually be added to better predict synergy. This integration could be accomplished by pre-processing multiple layers of information to derive a profile of gene scores for each cell line or tumor, which would then serve as input to PAIRWISE. As more studies are performed measuring the specific effects of anticancer drugs on the heterogeneous individual cells and subpopulations, PAIRWISE could be applied to these datasets to rapidly scale and identify novel combination and disease mechanism insights resulting in reduced bench-to-bedside timelines for patients. One major limitation of of PAIRWISE is that it is focused on synergy created by combined, simultaneous treatment with two drugs only. It does not model sequential treatment, where one drug creates a new molecular state that makes a second drug subsequently effective. It currently also does not predict higher-order combinations such as triplets or more. Incorporating these factors would require fundamentally different modeling approaches and relevant data, but will be necessary in the future. Our study include extensive validation data including extensive in vitro testing of PAIRWISE predictions in DLBCL, analysis of an existing PDX dataset showing that PAIRWISE-predicted synergy scores positively correlate with tumor response and improved progression-free survival. We however recognize that more systematic and comprehensive *in vivo* validation using PDX models would strengthen confidence in the clinical translatability of our predictions. Such in vivo data would be particularly valuable for prioritizing which of many predicted combinations warrant investment in clinical development, helping to bridge the gap between computational prediction and patient benefit. Ultimately, the goal of this framework is to provide a robust, experimentally-validated prioritization tool that can nominate the most promising combination therapies for prospective evaluation in clinical trials.

## Methods

### Curation of the gold standard drug combination training dataset and multi-modal data for feature extraction

To train the model, we harmonized data from five large drug combination screening resources: the NCI ALMANAC, ONEIL, MIT-MELANOMA, CLOUD, and the ASTRAZENECA-DREAM dataset, as well as eight relatively small drug combination screening datasets (Supplementary Table. 1). To obtain a sufficiently large drug combination dataset for model training, we curated the dataset for the uniform compound and cell line name entries. Among these data, triplets that are measured in different studies were compared and duplicate records where their synergy scores were averaged in the training dataset to reduce model over-fitting. The synergy scores (Loewe, Bliss, ZIP and HSA) were preprocessed and recomputed using the R package SynergyFinder v3.0. The gold standard drug combination screening data consisted of 279,452 cell line-drug combination triplets, covering 2,614 drug and 167 cell lines, termed the p13 dataset. All major tissue types were represented, with lung lineages the most prevalent.

To standardize drug chemical structures across datasets, we queried the PubChem entry via PUG REST for each drug name used in the p13 dataset to obtain an isomeric SMILES notation based on the drug name or InChIKey. Drugs with no matches in the initial search were manually annotated. In order to harmonize across multi-modal features including drug targets and their chemical fingerprints, we intended to use DrugBank ID as a unique identifier. Since there is no mapping dictionary between PubChem ID and DrugBank ID, we calculated a structural similarity profile of individual drugs based on one-hot vector representation (Supplementary Fig 1.). The structural similarity profile contains pairwise structural similarity scores between the input drug and all the available drugs from DrugBank which serves as an anchored comparison target. The similarity profile is generated for each drug present in the p13 dataset. Structural similarity between two drugs is measured by the Tanimoto index, which is defined as the number of common shared chemical fingerprints divided by the total number of the union of chemical fingerprints of the two drugs being compared. Beyond drug, we sought to standardize cell line name and its synonyms by obtaining its unique DepMap ID. We customized Python code via Cellosaurus API to retrieve a Cellosaurus research resource identifier (RRID) for each cell line name appearing in the p13 dataset. We transformed RRID to a static primary key assigned by DepMap to each cell line with a mapping file ‘Sample_info_v2.csv ‘, downloaded from DepMap Public 21Q3.

In machine learning, feature selection facilitates successful model building since the goal is to learn meaningful intrinsic relationships between input features and output targets. For predicting synergy between anti-cancer drug combinations in cell lines, input consists of both drug and cell line features that are predictive of drug synergy. Therefore we designed three modules to process model inputs and perform feature selection. This includes a chemical fingerprint module, drug target module, and cancer sample module. The ***chemical fingerprint module*** in PAIRWISE takes molecular graphs of drug molecules as inputs. This was done by converting the SMILE strings into molecular graphs with a Python package RDKit (version 2019). One variant model (PAIRWISE-Morgan) integrates alternative structural information to assess model prediction ability. PAIRWISE-Morgan takes the Morgan fingerprint (radius = 2) as inputs. Morgan fingerprint decomposes each chemical structure into molecular fragments by iteratively obtaining distinct paths through each atom of the molecule. These molecular fragments were hashed into a bit vector of length 2,048 to be used for model training.

In the ***drug target module***, the drug target profile was collected from Drug Target Commons (version 2.0), which is a cloud-based platform consisting of curated experimental drug-target interaction (DTI) data. The initial input for PAIRWISE is an interaction matrix of 2,614 drugs by 4,098 protein targets where binary values indicate whether a drug targets a protein. This inclusive approach captures both direct targets and proteins with experimental evidence of drug interaction, enabling a comprehensive representation of each drug’s biological activity profile. The 4,098 targets include primary pharmacological targets, off-targets, and proteins identified through various experimental methods (e.g., affinity chromatography, thermal proteome profiling). Our approach prioritizes coverage to ensure that the diverse mechanisms of action across 2,614 drugs are adequately represented in the model. A variant model, PAIRWISE-DrugBank, utilized drug target profile collected from Drugbank (5.1.10), resulting in a 2,614 drugs *2,401 target binary matrix. To create effective drug target features for our predictive model, we applied network propagation using protein-protein interaction data from STRING database on the binary DTI interaction matrix. This network propagation step (using STRING PPI data) enriches target information with biological context, transforming binary interaction data into a continuous representation that captures how drugs influence interconnected biological pathways. After matching the drugs to those in p13 dataset, we obtained a continuous interaction matrix of 2,614 drugs by 4,098 gene targets. We tested embedding sizes of 200, 500, 800, 1000, and 1200 dimensions to determine the optimal size. Although larger embeddings performed better on training data, they showed signs of overfitting (Supplementary Fig 12). Smaller embeddings performed poorly, lacking sufficient DTI information. We finally selected the 500-dimensional embedding, as it provided the best balance between accuracy and generalizability. To assess potential bias introduced by highly connected ‘hub’ proteins in the PPI network, we also conducted a sensitivity analysis comparing model performance using: (1) the full propagated network, (2) propagation after removing the top 5% most connected proteins, and (3) propagation with degree-normalized weights to reduce hub influence. The full network approach yielded the best cross-validation performance (AUROC = 0.847), compared to hub-removed (AUROC = 0.831) and degree-normalized (AUROC = 0.839) approaches, suggesting that hub proteins may carry important biological information about drug mechanism-of-action rather than merely introducing noise.

For the ***cancer sample module,*** we derived pre-treatment molecular signature from cell lines in the Cancer Dependency (DepMap) database. Transcriptome data (21Q4 Public, ‘CCLE_expression.csv’) was downloaded in the Cell Line Sample Info file (‘sample_info.csv’) from the DepMap portal. To identify a subset of potentially important genes that may contribute to drug synergy, we selected marker genes from four sources meant to effectively capture the heterogeneity of different samples: a landmark gene set comprising 978 genes from the LINCS project; the top 15% most differentially expressed genes in cancer (n = 3,101) for cancer cell lines in the CCLE project; furthermore, based on PPI network contained in the STRING database, we filtered interactions with a combined score higher than 0.7 and then identified the top 1000 protein genes that exhibit the most interactions with other proteins; finally a set of drug-target genes derived from the previously described binary matrix was also included. We combined the marker genes and the selection procedure yielded 4,079 genes, henceforth called ‘PAIRWISE marker genes’, and they were used for model construction.

### PAIRWISE neural network model.

We trained a multilayer neural network model to predict the synergy score of a cancer sample, e.g. a drug pair tested in a cancer cell line or a cancer patient tumor. The core of PAIRWISE modeling framework harnesses the power of the pre-training techniques to interpret and process three input features: chemical structure branch, drug target branch, and cancer sample branch. Each distinct features is initially transformed into a fixed-length vector using a modality-specific strategy, forming the initial for the feature fusion. A distinguishing strength of this framework lies in its integration of a cross-modal feature fusion module based on the transformer architecture. PAIRWISE acts to aggregate and employ a cross-attention mechanism on these vector inputs. This capability enhances the model’s robustness and predictive power, allowing it to adeptly simulate drug-cell, and drug-drug interactions, and achieve feature updating. The attention mechanism simulating the interaction of different modalities is characterized with the following equation,

$$Attention\left(Q,K,V\right)=softmax\left(Q*\frac{K^{T}}{\sqrt{d}}*V\right)$$

where *Q*(*queries*), *K*(*keys*), *V*(*values*) are generated matrix with the form of *XW*. *X* represents the input feature, and W is the learnable weights that linearly transform embeddings into *Q, K or V*. For example, to enable the module to reflect the effect of drug targets on cell PPI networks, our cross-attention module aims to find the most relevant embeddings based on attention weights between a combination of drug target embeddings, and cancer sample embedding. The updated five features (a combination of drug chemical structures, a combination of drug targets and individual cancer sample embeddings learned by PAIWISE) were flattened and concatenated to yield final embedding. The final output layer of a single neuron represents PAIRWISE predicted drug synergy probabilities, whose loss was measured against a binary synergy label. For each sample *i*, the output of the *j*+*1* layer *h_i_*^*(j*+*1)*^ is defined as a nonlinear function of the output of the *j*th layer *h_i_^(j)^* as follows:

hi(j+1)=Relu(Linear(hi(j)))

where Linear (*h_i_^(j)^*)) is linear function of *h_i_^(j)^* defined as *W^(j)^ x h_i_^(j)^* + *b^(j)^*. *W^(j)^* is the weight matrix and *b^(j)^* is the bias vector. Relu is the rectified linear activation function. The first layer *h_i_^(j)^* is the input drug chemical features, drug gene target features, and transcriptome features of sample *i* and the last layer *h_i_^(N)^* acts as its final prediction $\hat{p_{i}}\left(\theta \right)$ , where θ is a parameter containing W(j) and b(j) from all the linear layers. In order to train PAIRWISE, we scan all the parameters and determine the architecture of the neural network by cross validation. All parameters were updated by minimizing the binary cross-entropy loss. Therefore our *L* term of sample set, *C*, and parameters, θ , was :

$$L\left(C,\theta \right)=\frac{1}{M}\sum c_{i}\in c\left[p_{i}\log\left(\hat{p_{i}}\left(\theta \right)\right)+\left(\left(1-p_{i}\right)\log\left(1-\hat{p_{i}}\left(\theta \right)\right)\right)\right]$$

where *p_i_* is the observed binary synergy label for sample *i* and *M* is the number of samples in *C*.

### Model pre-training phase for multimodal data embeddings

In the pre-training phase, the aim is to train individual neural network module that can quickly adapt to a new drug or a new cell line/ tumor sample with only a few additional training samples, which is often the case when a new drug pair is going to be assessed in a new sample. The rationale is to acquire prior knowledge from a set of related tasks where training samples are particularly abundant. Each modality requires a specific method of embedding. we adopted an established computational framework with “unsupervised pre-training” design of transfer learning. The transfer learning design seeks to identify universal knowledge in each modality and then to transfer this knowledge to make robust predictions in a new condition. In the chemical fingerprint module, chemical PFM such as Grover has achieved state-of-art performance compared to other unsupervised learning techniques that were trained over millions of chemical compounds, and it is flexible such that it can be applied to drug’s 2D graph structure and output any drug’s chemical embedding. Each drug’s chemical structure was represented ’by an average of 256 activated bits in a single vector.

In the cancer sample module, we designed an autoencoder-based neural network (AE-CCLE) to generate robust transcriptomic embeddings. AE-CCLE was pre-trained on CCLE cell-line (n = 1,463) and TCGA tumor samples (n = 9,093), represented as log2(TPM + 1) values for 4,079 PAIRWISE marker genes. To specifically mitigate molecular noise inherent in TCGA data, we incorporated dropout regularization, systematically deactivating neuron subsets during training to reduce overfitting to noise. Rigorous internal cross-validation was conducted, reserving 10% of data for independent testing and performing 5-fold cross-validation on the remaining 90%. Following hyperparameter optimization, the best-performing autoencoder was fully trained (100 epochs), encoding transcriptomic data into informative, noise-resistant, 256-dimensional embeddings. These embeddings were combined with other modality-specific features for downstream PAIRWISE predictions.

We benchmarked AE-CCLE embeddings against other dimensionality-reduction techniques (PCA, ICA, and Random Projections) using qualitative (t-SNE visualization) and quantitative assessments (Silhouette Scores), confirming superior separation of tissue-specific signals by AE-CCLE. Further validation employed a Cox regression-based XGBoost survival prediction model, where AE-CCLE embeddings yielded the highest concordance-index (C-Index), indicating effective capture of clinically relevant signals rather than noise (Supplementary Fig. 13).

### Model assessment and data splitting strategies.

PAIRWISE and all comparison methods were assessed using a standard a five-fold cross-validation (CV) approach. During training, the model performance was evaluated on the validation set at the end of each epoch, and the model with the highest performance was selected. The performance of each model was measured using the area under the curve (AUROC) between actual and predicted drug synergy scores. We adopted three different methods of splitting the data for the CV folds, (1) leave-combo-out, (2) leave-drug-out, and (3) leave-sample-out. In the leave-combo-out approach, the cell line and a combination of drug triplets in the p13 dataset were divided into five folds of approximately equal size. For each fold in the cross-validation procedure, one fold of data was held out as the validation data, and the remaining four groups were pooled for training. The leave-drug-out and leave-sample-out approaches evaluate the model’s ability to predict drug combination synergy for previously unseen cell lines and compounds. This was done by splitting the drug / cell line into five clusters based on drug structure similarities / cell line transcriptome similarities. This procedure ensures that the test set did not include any drug or cell line previously present in the train set.

### Implementing benchmarked models with unified input features.

Prediction performances reported by the community submitted model usually cannot be used to directly compare between models, as they typically utilized data from different sources or applied different filtering or training-validation criteria. In order to provide insightful information in terms of prediction capabilities between benchmarked models, we conducted unbiased experiments using the same p13 dataset, and the unified input feature for drug representation and/or cell line representation as previously discussed in the multi-modal dataset for feature selection. Existing studies differ mainly in terms of regarding the synergy prediction as a regression problem, by directly predicting raw synergy scores, or as a classification task, by predicting binarized labels based on pre-defined thresholds on the specific synergy scores. We selected the Loewe score (threshold for 0) as training label for comparing binary classification capabilities (synergy or non-synergy) among the benchmark models.

Previous studies showed the machine learning features that consider both drug target information and molecular profiles would improve prediction performance. We evaluated eleven representative models which utilized different feature encoders to represent different types of input features, including 5 state-of-art methods (DeepSynergy, DeepDDS, MatchMaker, MultitaskDNN, Transynergy) and 6 variant models of PAIRWISE to assess the importance of each modality. In the replacement of cancer sample branch, PAIRWISE-PathwayNN is designed to simulate the effects of drugs on cells at the pathway level to reflect the underlying signaling mechanisms. For data preparation, for each of the cancer cell lines, we computed pathway enrichment scores for 1329 canonical pathways from MSigDB. We formulated the drug combination prediction task with pathway enrichment scores, in tandem with two drugs’ descriptors as a binary classification problem. PAIRWISE-PPI model combines pharmacology networks and complex biological networks. Given a cell line graph, we used Graph Attention Network (GAN) to update gene features. Cell line graph-level representations are concatenated with drug features, and then fed to the fully connected layer to predict synergy scores.

### Independent validation of DLBCL high-throughput combinatorial screening dataset

We obtained drug screening data that identifies drugs that cooperate with ibrutinib to kill DLBCL lymphoma cells from Project Tripod from NCATS, a data-sharing platform established by the National Center for Advancing Translational Sciences. This resource contains thousands of combination pairs that can be quickly narrowed down to identify the most effective pairs for follow-up and clinical studies. We removed compounds from the dataset without known drug target or chemical structures information. Transcriptome data of TMD8 DLBCL cell line (GSE93985) was obtained from the GEO database. Such filtering left 214 ibrutinib anchored combination assays in TMD8. We estimated the expected drug combination response using the full dose-response matrix, and the PAIRWISE predictions were compared to a single synergy score.

### Translation to DLBCL patient tumors

We obtained transcriptome data and clinical information for 562 DLBCL patients. For the survival analysis, we selected all untreated patients with clinical outcome data who received immunochemotherapy (R-CHOP or CHOP-like chemotherapy; 229 samples). PAIWISE, which did not use clinical information for training, was locked down before the analysis of clinical data, allowing us to analyze the relationship between hierarchical clustered subtypes and survival in the entire cohort.

For each DLBCL patient tumor, their genomic features were used as input to our machine learning model to predict the response to BTKi that our model had not previously seen, which altogether resulted in 268 pairwise combinations with 211 drugs from diverse target classes from NCATS MIPE library and 57 additional investigational drugs. We predicted patient response to each combination using our model pretrained on cell lines and the transcriptome profiles of each patient. We considered a combination to be “effective” if the model’s predicted drug synergy score is over 0.5. Then we performed unsupervised hierarchical clustering, which identified groups with similar characteristics. We classified a patient as BTKi Combo (+) or BTKi Combo (−) if they were in the distinct subgroups with different patterns of combination scores. We used a log-rank test (p<0.05) to determine the significance of the associated treatment outcomes (overall survival).

### Pathway analyses

To analyze the biological processes relevant for combinations containing BTKi in the dataset, we performed differential expression analysis. Raw RNAseq count data for the BTKi Combo responsive (+) and BTKi Combo non-responsive (−) were utilized. Raw counts were transformed to log2 counts per transcripts (log-TPM) and genes with low expression levels were removed (TPM < 0.01) as previously described. Differential expression was determined by using the linear model limma. We used the set of pathways from the Molecular Signature Database (MsigDB)^47^ or the lymphoid signature database^48^for enrichment tests. We identified genes that were significantly differentially expressed when contracting the BTKi Combo (+) and BTKi Combo (−) cell lines. FDR correction was applied using the Benjamini-Hochberg procedure. We also conducted pathway enrichment analysis to compare predicted synergistic and non-synergistic BTKi drug combinations in DLBCL. Gene targets from the top 30 synergistic combinations (median predicted probabilities > 0.5) and 30 randomly selected non-synergistic controls( 0 ≤ predicted probabilities ≤ 0.5 ) were pooled separately. Each gene set underwent KEGG and GO enrichment analysis using DAVID v6.8, with multiple testing corrected via Benjamini–Hochberg FDR (q<0.05).

### High-throughput drug combination screening

Lymphoma cell lines including ABC-DLBCL cell lines: U2932, OCI–LY3, SUDHL–2, SUDHL–10, OCI–LY10, TMD8: GCB-DLBCL cell lines: FARAGE, SUDHL–4 were generously donated by the Melnick lab at Weill Cornell Medicine. Cell lines were cultured in basal medium [DMEM (Gibco) supplanted with 10% FBS and 1% penicillin/streptomycin (P/S)] and maintained in a 37° incubator at 5% CO2. The HTS drug-screening library composed of 30 targeted-compounds were purchased from MedchemExpress, and a subset of them were used as AstraZeneca ‘investigational drugs. Drugs were selected based on current clinical applications (FDA approved), selectivity (target of canonical signaling pathways JAK/STAT, Ras/ERK, PI3K/ATK, anti-apoptotic etc.) and redundancy (multiple drugs targeting the same pathways). Collectively a total of 21 proteins we targeted.

The high-throughput screen was conducted using a fully integrated HighRes Biosolutions automation platform, controlled by Cellario software. The system incorporated a Hamilton Microlab Star liquid handler, a Prime Automated Liquid Handler, a HighRes Biosolutions Microspin plate centrifuge, plate incubators, and a Biotek Synergy H4 plate reader.

Prior to combination screening, the single-agent activity of the drug library (MedChemExpress) was assessed to determine cell line-specific synergistic effects. Results from this single-agent screening indicated that a dose range between ~10 nM and 10 μM would capture both active and inactive concentrations. The combination experiments utilized a 6x6 matrix block design, with customized starting concentrations and serial 1:3 dilutions for each agent, handled by the Hamilton Microlab Star.

Lymphoma cell lines were seeded in 384-well plates at a density optimized for each cell line’s proliferation, using the Prime Automated Liquid Handler. Cells were incubated for 96 hours in the presence of the drug combinations. Following incubation, the assay readout was performed using CellTiter-Glo^®^ (Promega) reagent, following the manufacturer’s protocol. Luminescence was measured using the Biotek Synergy H4 plate reader.

Quality control was ensured by monitoring the coefficient of variation (CV) of the DMSO control wells, with an acceptable CV threshold of <2%. Data analysis, including processing and visualization, was carried out using PAIRWISE Explorer.

### Key modules of benchmark platform

Since we want to evaluate which model architectures can potentially correspond well to the true biological relations between selected sets of features and ‘predictive performance’, we curated a gold-standard p13 dataset with the search space of more than 200,000 drug combinations with unified identifiers for drugs and cell lines, far beyond any individual release of dataset reported by each model in the original paper. The benchmark platform allows users to (i) load preset or customized drug combination data from a local file; (ii) specify the type of representation methods for drug and cell lines; (iii) split the dataset into training, validation and testing sets; (iv) initialize a machine learning model or load a pre-saved model with configuration files containing saved model architecture and parameters. (v) train the model and monitor the progress of training and performance metrics.

### End-to-end pipeline to compare PAIRWISE model with other models.

First, we curated ground-truth datasets for both cell-line and clinically tested drug combinations. Second, we develop PAIRWISE model and re-construct other drug combination prediction models using the same drugs and transcriptome inputs. Our pipeline is capable of modeling heterogeneous data at multiple scales. Third, we benchmark PAIRWISE model with other community submitted methods related to the discovery and development of effective combination therapies. Through extensive experiments on selected datasets, we demonstrated our model has attained superior predictive performance and generalization ability to novel data points. Additionally, our pipeline offers a range of data functions including various types of molecule feature generation, strategies for systematic model evaluation, and interactive visualization for comparison and exploration, all of which are integrated and accessible through an open Python library. Last, we apply PAIRWISE model to a dataset of ex vivo anticancer drug synergy measurements for patients with DLBCL, identifying not only biological processes that are important for the determination of drug synergy but also uncovering clinical benefits on the sub-groups of patients that are useful for personalized drug combination recommendations.

We implemented an end-to-end pipeline to assist the development and evaluation of drug combination prediction models (Supplementary Fig. 2). Next, to benchmark PAIRWISE with state-of-the-art methods, our pipeline incorporated PAIRWISE along with 5 previously published methods predicting responses of combination treatments and 6 variant PAIRWISE models with distinct drug chemical, drug target, and cancer representations (Supplementary Table. 2). All these models were developed using the same transcriptome profile or drug chemical datasets as the PAIRWISE model despite their unique feature engineering purpose. Third, our pipeline enables users for systematic evaluation and comparison among different methods using the newly curated database. The prediction results of each model can be accessed through the website (Code availability). Finally, we applied PAIRWISE into real-world scenarios, with patient tumors or drug compound that were never seen before by the model.

### Clinical-Trial Validation of PAIRWISE Predictions

We downloaded the Drug Combination DataBase (DCDB version 2.0; downloaded on 1 May 2025; http://www.cls.zju.edu.cn/dcdb/), which aggregates records from the FDA Orange Book and ClinicalTrials.gov, to assemble a set of clinically evaluated oncology combinations. Four sequential filters were applied. First, any entry whose trial description explicitly stated “no benefit” or “no improvement” was removed. Second, records were restricted to neoplasm-related ICD-10 codes (C34, C34.90, C64, C64.9, C50, C50.9, C79.81†, C91, C91.00, C91.1, C91.5, C92, C92.00, C92.1, C92.40, C92.6, C94.20, C94.3, C95, C56, C61, C43, C43.9, C18, C18.9, D01.0), eliminating non-oncology indications. Third, both agents in every surviving pair had to possess a DrugBank identifier, a canonical SMILES string and at least one annotated molecular target so that PAIRWISE could compute its full chemical–biological feature vector. Fourth, disease descriptors were mapped to The Cancer Genome Atlas (TCGA) primary-site labels through MeSH-based fuzzy matching followed by manual review. After filtering, 196 unique drug combinations were retained. These pairs were merged with bulk-tumor RNA-seq profiles from TCGA, and PAIRWISE predicted synergy probabilities for each combination in every individual tumor. Probabilities were then averaged within each cancer type to obtain a tissue-level synergy score. To provide a reference distribution, we generated a background set of randomly selected, untested drug pairs drawn from the same compound pool. Within each tissue, the distribution of PAIRWISE scores for clinically tested pairs was compared with that for the background set using a two-sided Kolmogorov–Smirnov test.

## Supplementary Material

1

This is a list of supplementary Files associated with this preprint. Click to download.

• SupplementaryTable5BTKirawdoseresponseandcalculatedsynergyscores.xlsx

## Figures and Tables

**Table 1.** Performance comparison of PAIRWISE to benchmarked models

Method	Leave-combo-out		Leave-drug-out		Leave-sample-out	
	AUROC	AUPRC	AUROC	AUPRC	AUROC	AUPRC
**PAIRWISE**	**0.855±0.004**	**0.710±0.005**	**0.847±0.006**	**0.702±0.004**	**0.822±0.003**	**0.670±0.004**
Transynergy	0.806±0.005	0.612±0.003	0.787±0.004	0.591±0.005	0.770±0.005	0.572±0.004
Multitask-DNN	0.762±0.006	0.559±0.004	0.734±0.003	0.537±0.005	0.729±0.007	0.510±0.006
DeepSynergy	0.725±0.008	0.510±0.005	0.720±0.006	0.580±0.006	0.712±0.007	0.577±0.007
DeepDDS	0.688±0.006	0.473±0.005	0.652±0.005	0.438±0.006	0.654±0.007	0.480±0.008
Matchmaker	0.687±0.007	0.473±0.006	0.645±0.006	0.420±0.007	0.640±0.004	0.435±0.004

**Table 2.** Spearman correlation between predicted synergy probabilities and experimental synergy scores in three settings

Method	Leave-combo-out	Leave-sample-out	Leave-drug-out
	rho	rho	rho
**PAIRWISE**	**0.626±0.004**	**0.646±0.004**	**0.595±0.002**
Transynergy	0.574±0.002	0.532±0.002	0.480±0.003
Multitask-DNN	0.497±0.004	0.440±0.004	0.403±0.005
DeepSynergy	0.423±0.003	0.394±0.004	0.368±0.004
DeepDDS	0.304±0.003	0.244±0.006	0.247±0.003
Matchmaker	0.300±0.007	0.229±0.006	0.214±0.005

**Table 3.** Performance comparison between PAIRWISE and variant models

	Models	Leave-combo-out		Leave-cell-out		Leave-drug-out	
		AUROC	AUPRC	AUROC	AUPRC	AUROC	AUPRC
**Original model**	**PAIRWISE**	**0.855±0.004**	**0.710±0.005**	**0.847±0.006**	**0.702±0.007**	**0.822±0.003**	**0.670±0.004**
Variant Chemical Fingerprint module	PAIRWISE-SMILES	0.769±0.012	0.321±0.010	0.763±0.005	0.321±0.008	0.755±0.006	0.285±0.005
	PAIRWISE-Morgan	0.779±0.010	0.336±0.008	0.769±0.005	0.318±0.003	0.766±0.004	0.276±0.007
Variant Drug Target module	PAIRWISE-DrugBank	0.800±0.007	0.673±0.008	0.805±0.010	0.673±0.008	0.792±0.006	0.593±0.003
	PAIRWISE-PropagatedDrugBank	0.812±0.005	0.690±0.005	0.810±0.007	0.661±0.006	0.813±0.004	0.664±0.006
Variant Cancer Sample module	PAIRWISE-PPI	0.801±0.011	0.618±0.007	0.794±0.006	0.606±0.012	0.80±0.006	0.617±0.004
	PAIRWISE-GSVA	0.776±0.008	0.578±0.005	0.754±0.003	0.549±0.014	0.747±0.004	0.544±0.005

## Data Availability

PAIRWISE and the benchmarking platform can be downloaded from GitHub (https://github.com/Mew233/pairwise). Prediction results of PAIRWISE and all benchmarked models can be accessed on ShinyApp (https://mew233.shinyapps.io/synergyy_shinyr/). BTKi drug combination experimental data in DLBCL can be accessed through PAIWISE explorer (https://mew233.shinyapps.io/PAIRWISE_Explorer/).
