# Supplementary material for: PAIRWISE: Deep Learning-based Prediction of Effective Personalized Drug Combinations in Cancer
Source: Res Sq. 2026 Jan 19:rs.3.rs-8518203. Preprint. [Version 1] doi: 10.21203/rs.3.rs-8518203/v1 (PMC12869695; doi:10.21203/rs.3.rs-8518203/v1)
Supplement: 1 [file NIHPPrs8518203v1-supplement-1.pdf]

Supplementary text

PAIRWISE Identifies Novel Lineage-Specific and Multi-Cancer Synergistic Combinations in an Independent PDX Dataset.

To investigate PAIRWISE's capability to uncover novel pan-cancer and lineage-specific synergistic drug combinations, we conducted an analysis using an independent patient-derived xenograft (PDX) dataset[49] . This dataset, encompassing 399 tumor samples across 12 distinct tumor types (including breast carcinoma, non-small cell lung carcinoma, colorectal cancer, and pancreatic ductal carcinoma), was particularly suitable for this purpose. Its breadth in tumor types and, critically, the inclusion of drug combination tests involving CDK4/6 inhibitors, allowed for a robust assessment of PAIRWISE's predictive performance in a new context.

Utilizing PAIRWISE, we predicted the synergy of CDK4/6 inhibition (specifically, ribociclib) when combined with a library of 812 other drugs across these PDX samples (**Supplementary Fig. 14a**). To substantiate these *in silico* predictions, we focused on *in vivo* response data available for breast cancer PDXs within the Gao et al. dataset. Our analysis revealed a significant positive correlation (Pearson's Coefficient $r=0.388$, $p<0.001$) between PAIRWISE-predicted synergy scores and the actual tumor responses observed in these PDX models (**Supplementary Fig. 14b**). Furthermore, breast cancer PDX samples that PAIRWISE predicted would respond synergistically to CDK4/6i drug combinations demonstrated significantly longer

progression-free survival compared to those treated with combinations predicted to be non-synergistic (log-rank p=0.006, **Supplementary Fig. 14c**). This finding suggests that the synergistic status predicted by PAIRWISE was associated with delayed tumor progression *in vivo*.

Our broader analysis of all predicted synergistic combinations across the 12 tumor types indicated a distinct distribution of tissue specificity (**Supplementary Fig. 14d**). Approximately 62% of the combinations predicted to be synergistic were lineage-specific, meaning their predicted efficacy was confined to individual cancer types. The remaining 38% exhibited broader predicted multi-cancer activity, showing synergy in more than one tissue type. While no CDK4/6 inhibitor combinations were predicted to be synergistic across all 12 tissue types evaluated, we identified three combinations with predicted synergy across eight different tissues, highlighting a significant multi-cancer potential. For instance, the combination of ribociclib with compounds such as IPA-3 and caffeic acid phenethyl ester demonstrated notable potential for broad multi-cancer synergy.

Collectively, this analysis of an independent PDX dataset underscores PAIRWISE's ability not only to predict synergistic combinations with *in vivo* relevance but also to identify both novel lineage-specific and broader multi-cancer therapeutic opportunities.

Supplementary figures

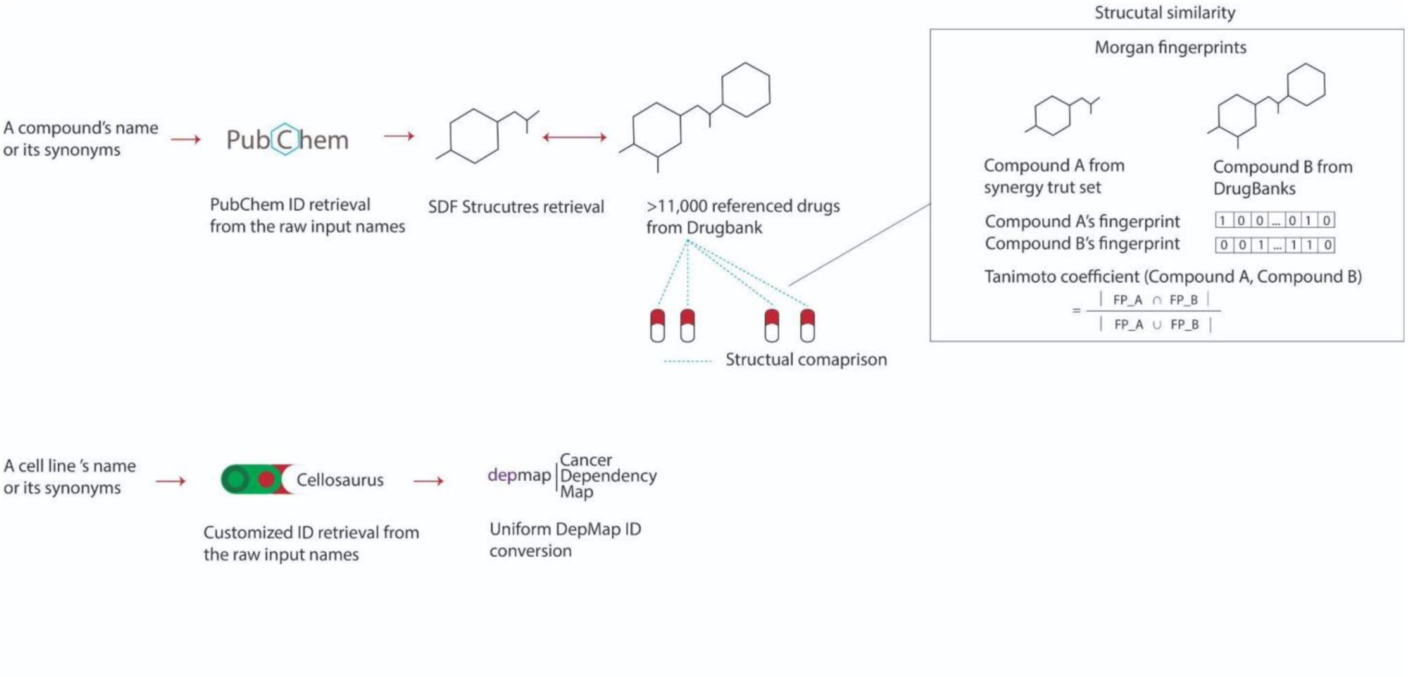

Supplementary Fig. 1 Retrieval, harmonization, and curation of drug combination datasets (p13).

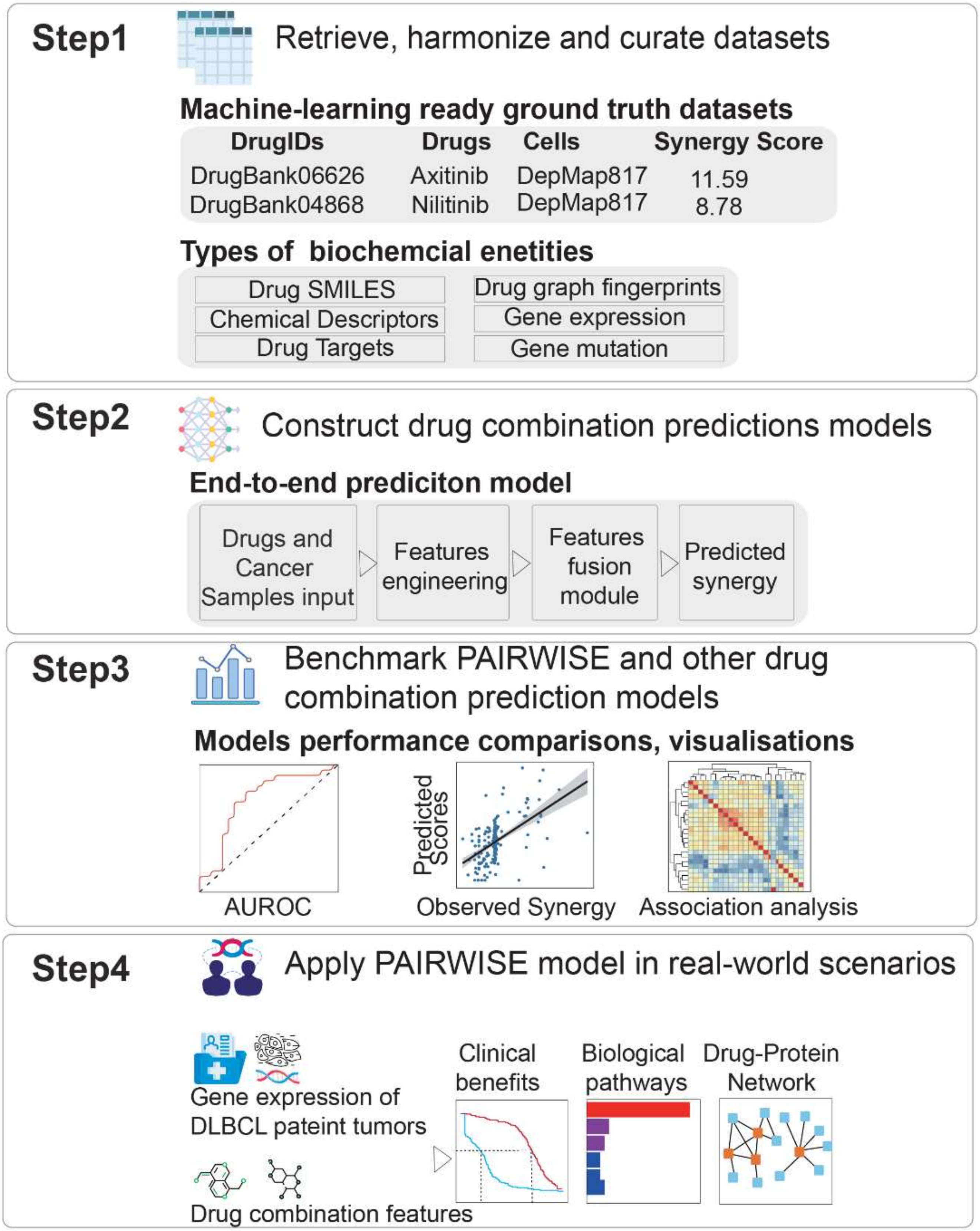

Supplementary Fig. 2 End-to-end pipeline to compare PAIRWISE model with other models.

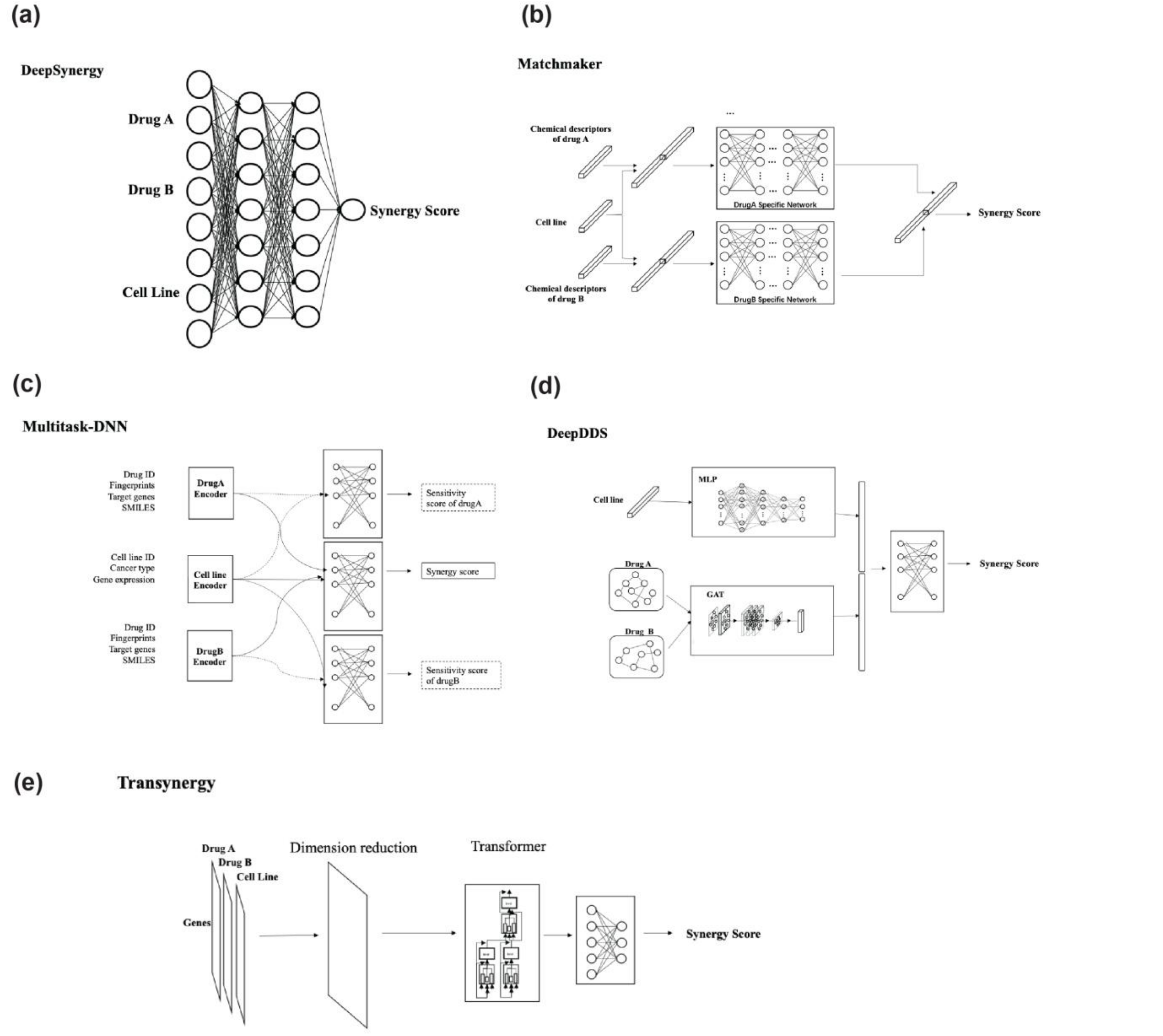

Supplementary Fig. 3 Overview of the synergy prediction deep learning architectures that were compared to PAIRWISE (a) the DeepSynergy computational approach. The feed-forward neural network integrates gene expression profiles of cell lines and chemical descriptors from two drugs. Concatenated vectors comprising these features form the input layer. Data are processed through hidden neural layers, culminating in a single output neuron predicting the synergy score. **(b) the MatchMaker computational approach.** Drug-specific subnetworks (DSNs) independently process the combined chemical descriptors of each drug with the gene expression profile of the cell line to generate drug-specific representations. These learned representations are subsequently integrated by multiple fully connected layers, which outputs the predicted Loewe synergy values. The network is optimized through an end-to-end training strategy to minimize a weighted mean squared error (MSE) loss. **(c) the Multitask-DNN computational approach.** Drug encoder learns an embedding representation of a drug. Inputs are MACCS fingerprints, canonical SMILES,and target genes. Cell line encoder learns an embedding representation of a cell line. Inputs are cancer type and gene expression profile. Sensitivity is an auxiliary output to augment synergy prediction. **(d) the DeepDDS computational approach.** Initially, chemical structures of drugs are modeled as graphs with atoms as nodes and chemical bonds as edges. These graphs are processed using graph convolutional networks and attention mechanisms (GATs) to generate drug embeddings. Gene expression profiles of cancer cell lines are represented via Multi-Layer Perceptrons (MLPs). The resulting embeddings from both drugs and cell lines are concatenated and subsequently passed through fully connected layers to predict drug synergy effects. **(e) the Transynergy computational approach.** The architecture comprises three main components: an input dimension reduction network, a transformer-based self-attention module, and a fully connected output network. Drug-target interaction profiles and cell line gene expression or dependency data form the initial input, organized into a gene-by-feature matrix. The dimension reduction layer condenses these high-dimensional inputs, which then feed into a transformer component specifically designed to capture gene-gene interactions through self-attention mechanisms. Finally, the learned representations are processed by fully connected layers to produce the synergy score.

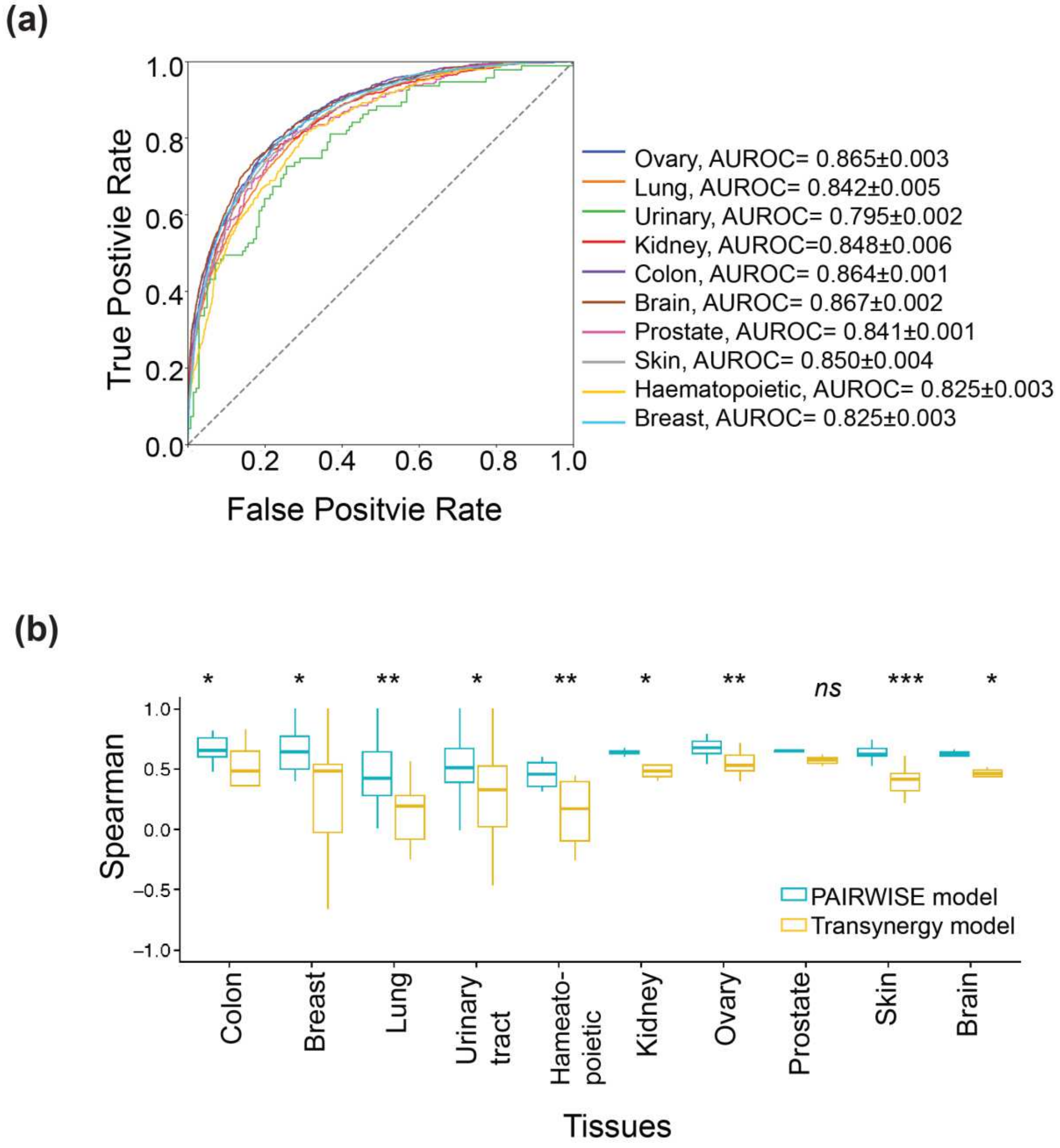

Supplementary Fig. 4 (a) PAIRWISE exhibited consistent AUROC performance across tissues. **(b)** Spearman correlation coefficients of the PAIRWISE predictions and Transynergy predictions across cell lines from different tissues of origins

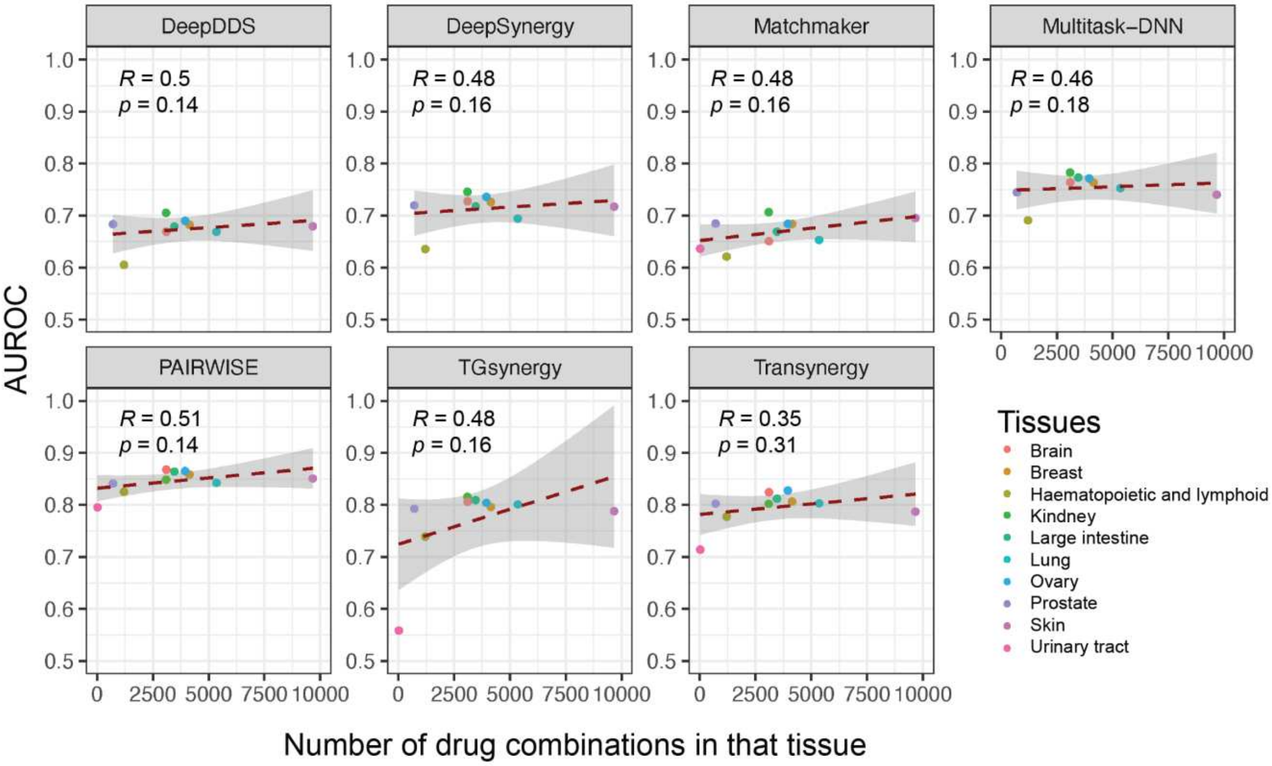

Supplementary Fig. 5 The correlation between model performance with AUROC as metrics and number of drug combinations in that tissue for all benchmark models.

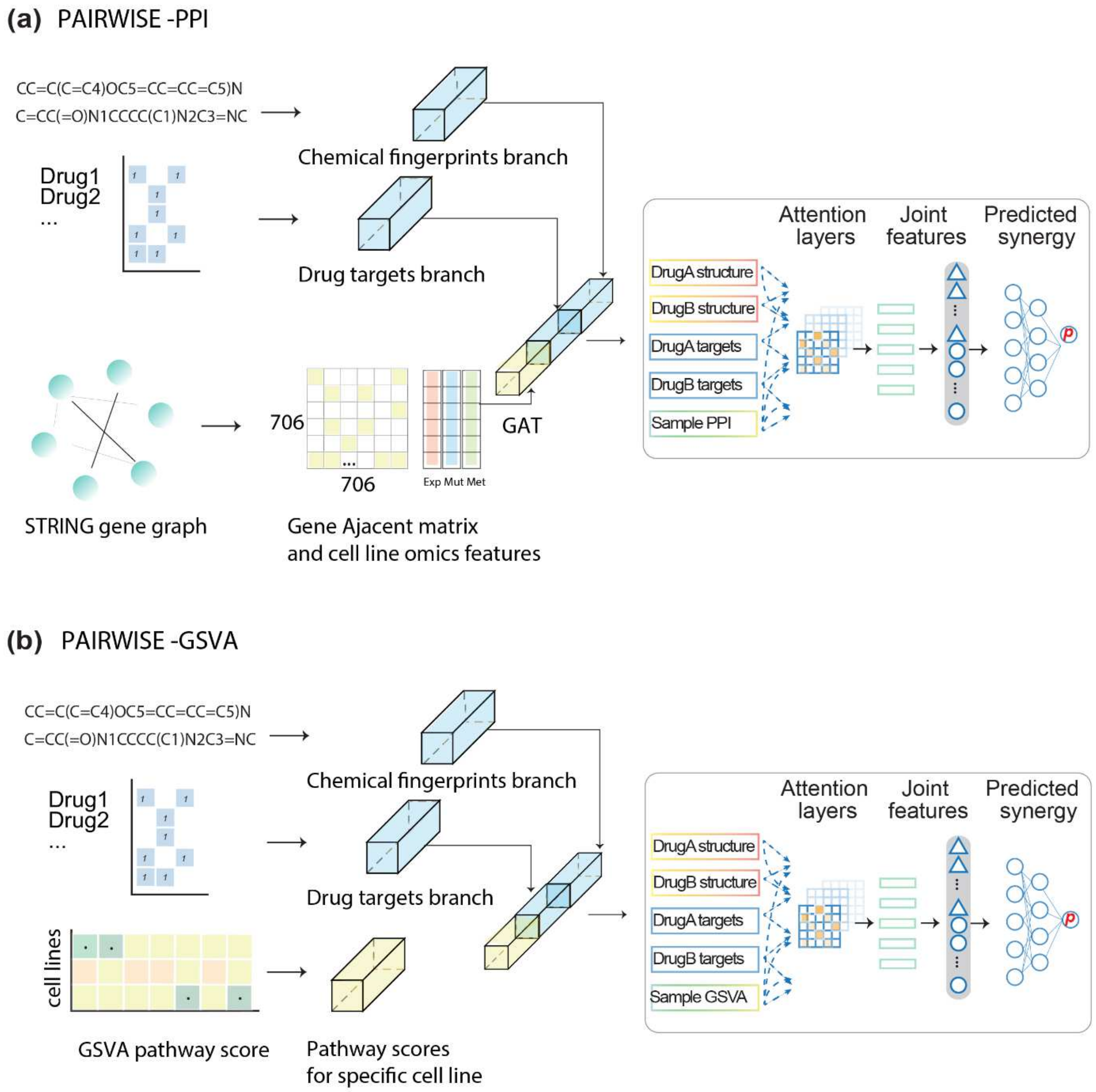

Supplementary Fig. 6 Variant models of PAIRWISE. (**a**) The cell line representation module was derived from PPI network using a GAT structure. **(b)**The cell line representation module was derived with GSVA pathway scores per cell line.

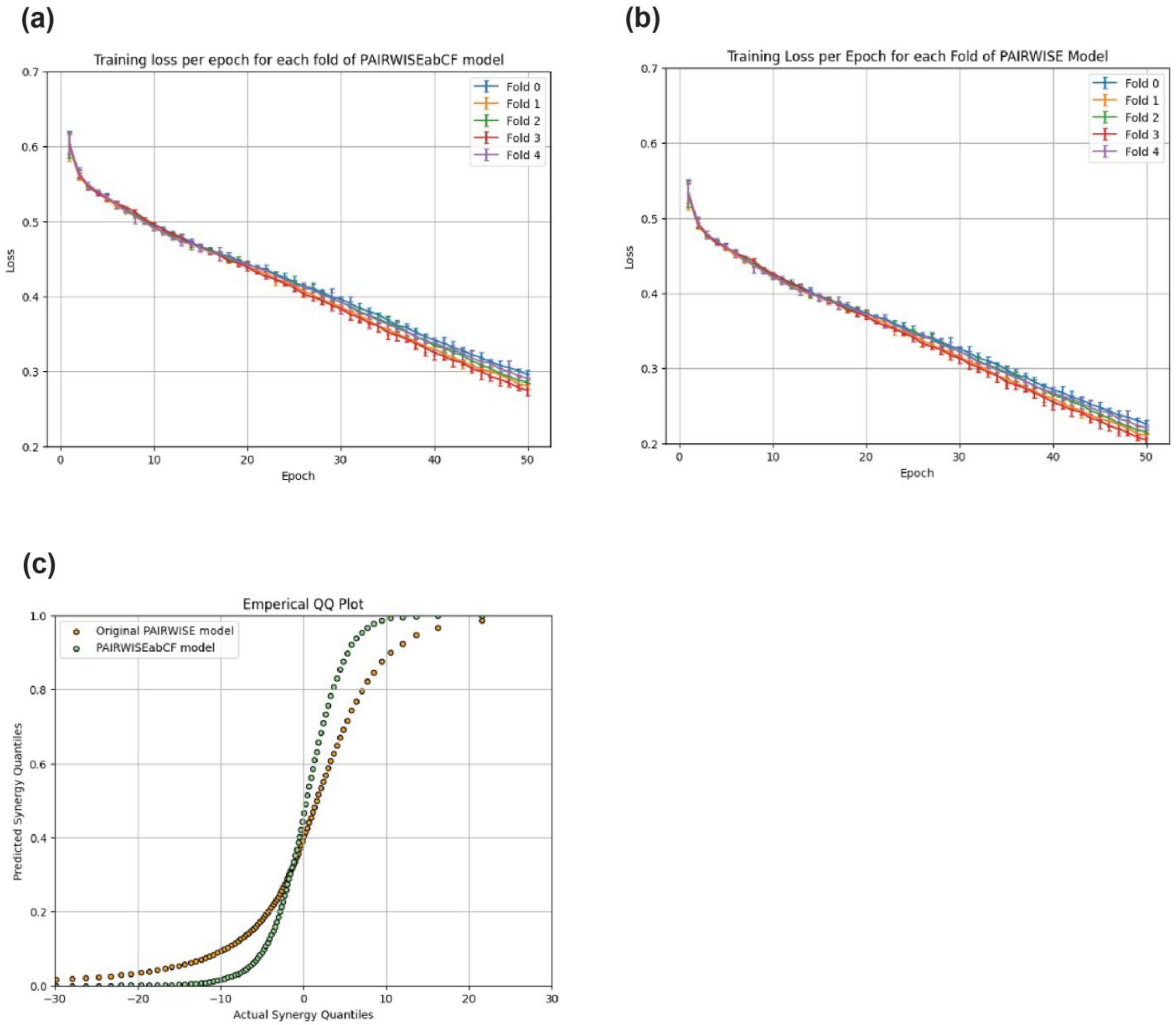

Supplementary Fig. 7 (a, b) Training loss per epoch for each fold of PAIRWISEabCF (a) and PAIRWISE (b) model. The initial loss of PAIRWISE model is 0.6 while the initial loss of PAIRWISEabCF model is 0.55. During the epochs of training, we observed the training loss of both models steadily decreased, indicating both models converge their predictions to the ground truth. At the epoch 50, the loss of PAIRWISE model is 0.2 while the loss of PAIRWISEabCF model is 0.3, showing that PAIRWISEabCF had more erroneous predictions during the training than PAIRWISE. (**c**) Quantile-quantile plot modeling the relationship between observed and expected synergy scores. Actual synergy quantiles were observed synergy scores from the experimental Loewe scores, while predicted synergy quantiles were predicted synergy scores. PAIRWISEabCF predictions were skewed toward extremes, suggesting overfitting on synergistic and non-synergistic labels (actual synergy scores between -20 and 20), leading to reduced generalizability and poorer performance.

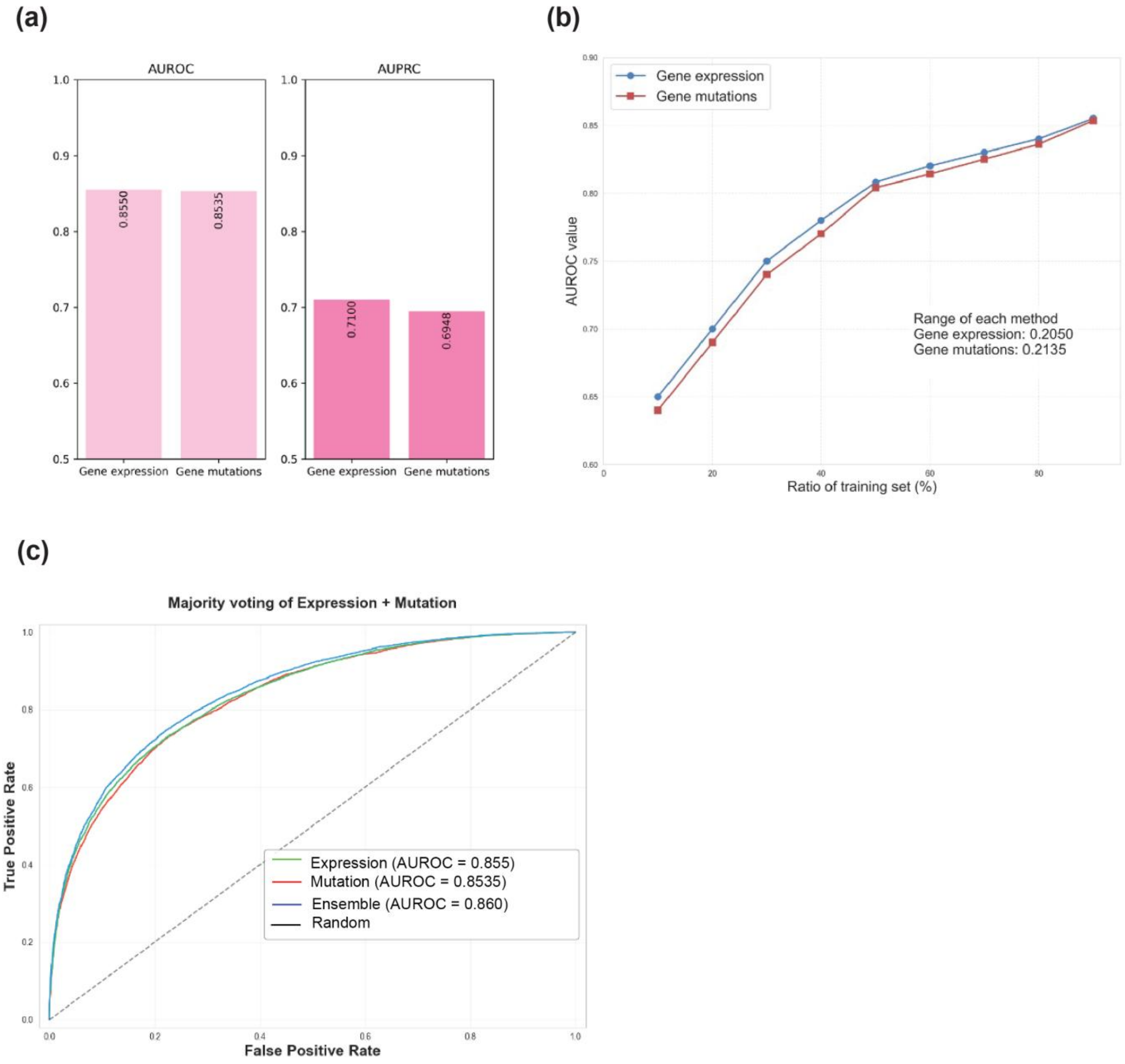

Supplementary Fig. 8. (a) Performance of PAIRWISE-Mut and PAIRWISE models across metrics including AUROC, and AUPRC. PAIRWISE consistently outperformed Mut-PAIRWISE in all metrics. **(b).** AUROC value of Mut-PAIRWISE and PAIRWISE with increased training set size. **(c)** An ensemble (expression+mutation) model achieves marginal performance improvement over PAIRWISE and PAIRWISE-mut.

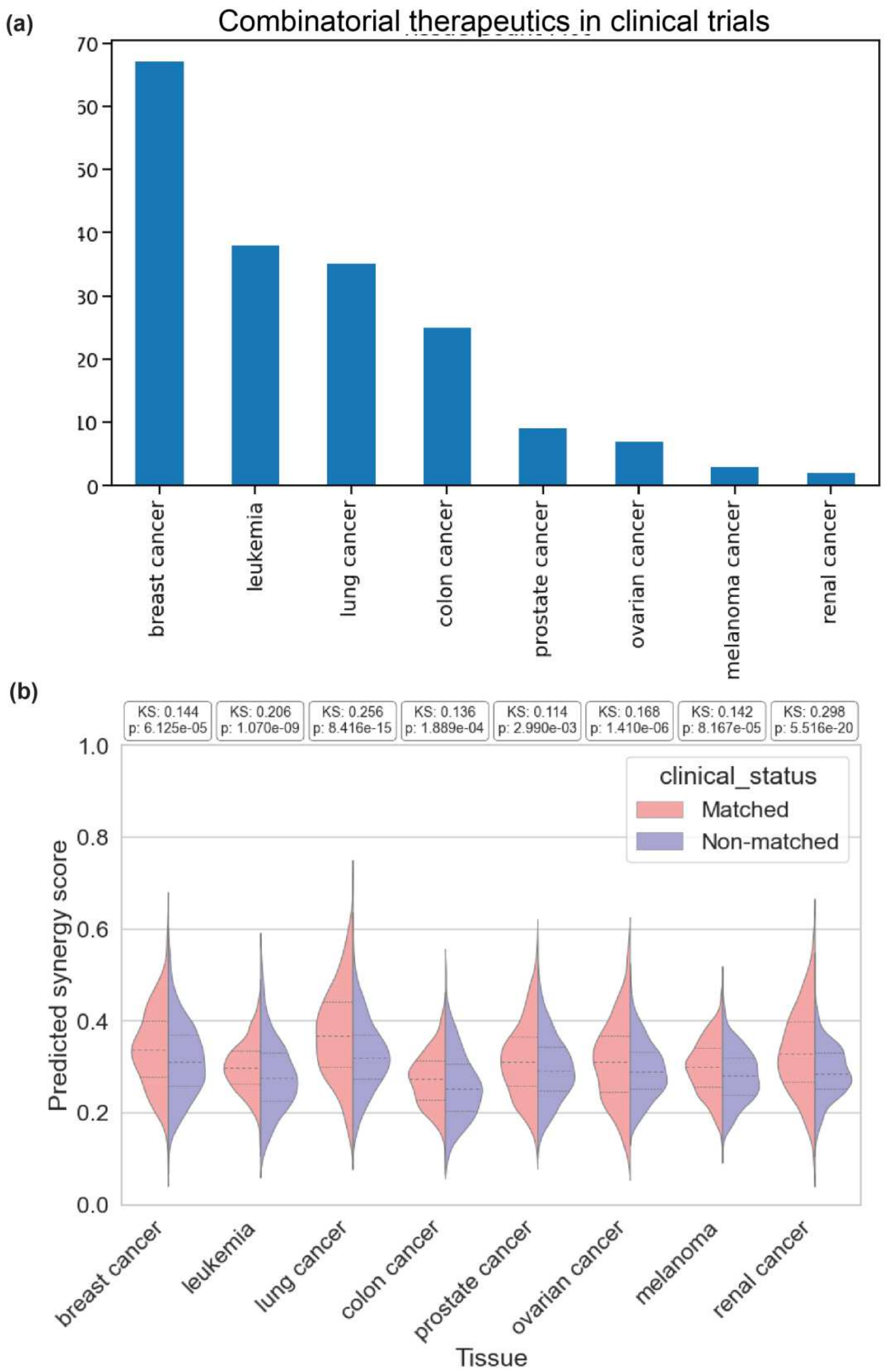

Supplementary Fig. 9. Analysis of drug combinations in clinical trials. (a) Distribution of clinically synergistic drug pairs in each tissue/cancer type. **(b)** The clinical importance and performance of PAIRWISE. Violin plots of the distribution of synergy scores, predicted by PAIRWISE, for drug pairs That have been clinically tested and those that have not. The confidence interval for the median score of both clinically tested and non-clinically tested pairs are shown in each tissue. KS tests were used to find the d-statistic and p-value.

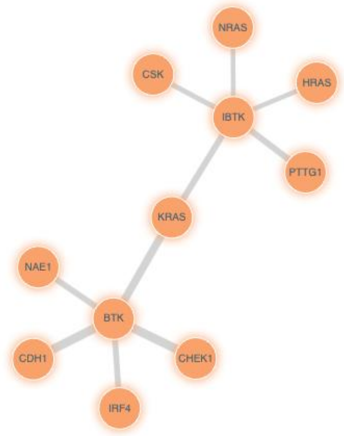

SL Graph

Supplementary Fig. 10 BTK combination targets synthetic lethal pairs of genes as defined by SynLethDB including CHK1, Prexasertib is such as a CHK1 inhibitor tested in our experiment.

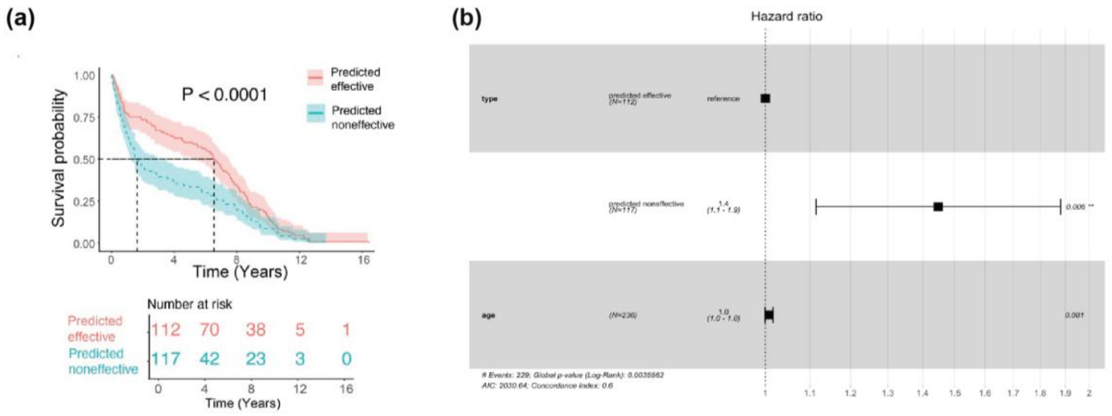

(c)
```
> coxph(formula = Surv(Progression_Free.Survival._PFS_.Time._yrs) ~ type, data = metadata)

                    coef   exp(coef) se(coef)    z        p
type              0.3815   1.4644    0.1333   2.862   0.00421

Likelihood ratio test=8.17  on 1 df, p=0.004256, n= 229, number of events= 229
```

(d)
```
> coxph(Surv(Progression_Free.Survival._PFS_.Time._yrs) ~ type+age, data = metadata)

              coef       exp(coef)        se(coef)          z        p
type      0.369988  1.447717         0.133482          2.772    0.00557
age       0.007654  1.007684         0.004391          1.743    0.08132

Likelihood ratio test=11.28  on 2 df, p=0.003556, n= 229, number of events= 229
```

Supplementary Fig. 11: (a) Differences in survival upon R-CHOP between BTKiCombo(+) (Predicted effective) and BTKiCombo(-) (Predicted noneffective) tumors. P-value was calculated using Cox proportional hazard analysis. **(b)** Forest plot for the BTKi-Combo (+) vs. BTKi-Combo (-) on PFS among DLBCL patients. Comparisons of the survival curves in Fig 5b were performed with a two-sided log-rank test. P values reported in this plot are two-tailed from Cox proportional hazard regression analyses. Black square represents the HR value. Error bars represent the 95% confidence intervals. PAIRWISE predicted score was associated with worse PFS in the predicted DLBCL BTKi-Combo (-) group (HR = 1.4 (1.1-1.9), P < 0.006). By contrast, age did not appear to have any effect on PFS (HR = 1 (1.0=1.0), P = 0.081). **(c-d)** Multivariate Cox analysis for the BTKi-Combo (+) vs. BTKi-Combo (-) patients, and with consideration of covariates age. The covariate of PAIRWISE identified type remains significant (p < 0.05). However, the covariate age is not a significant contribution (coef =0.007, p = 0.08).

(a)

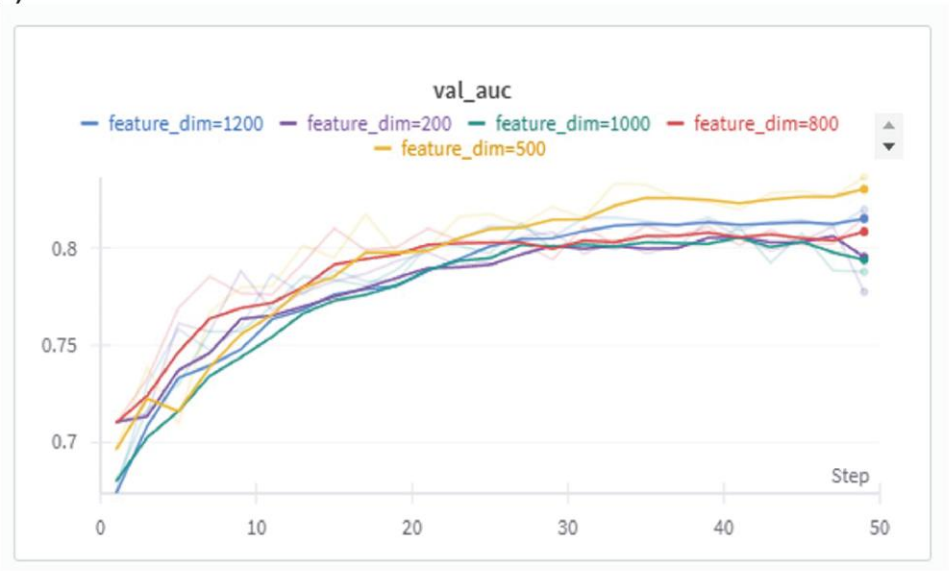

(b)

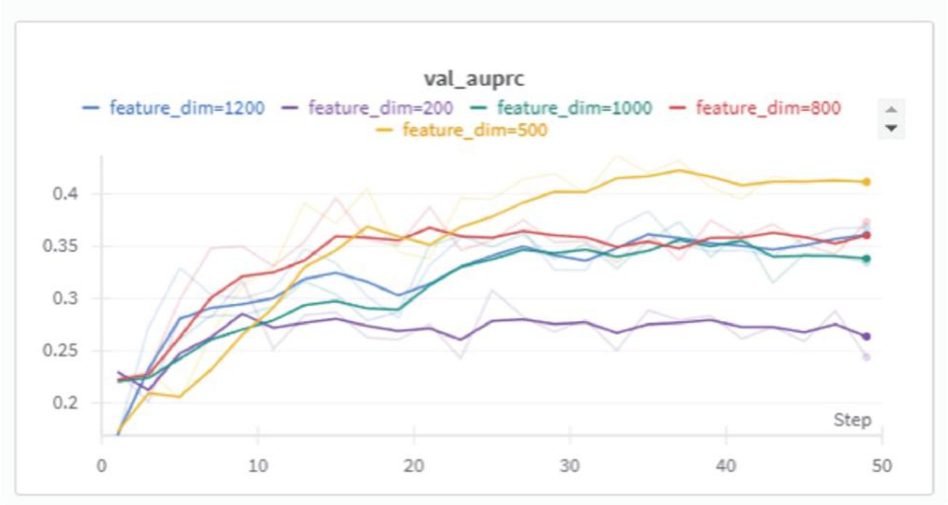

Supplementary Fig. 12. Performance metrics of training and validation across varying embedding dimensions. Model performance (a) AUROC and (b) AUPRC on validation datasets was systematically evaluated across embedding sizes (200, 500, 800, 1000, and 1200 dimensions). Optimal predictive performance on validation data was achieved at 500-dimensional embeddings, balancing model complexity and generalizability. Higher dimensions (>800) demonstrated signs of overfitting, whereas lower dimensions (200) underperformed, indicating insufficient representational capacity.

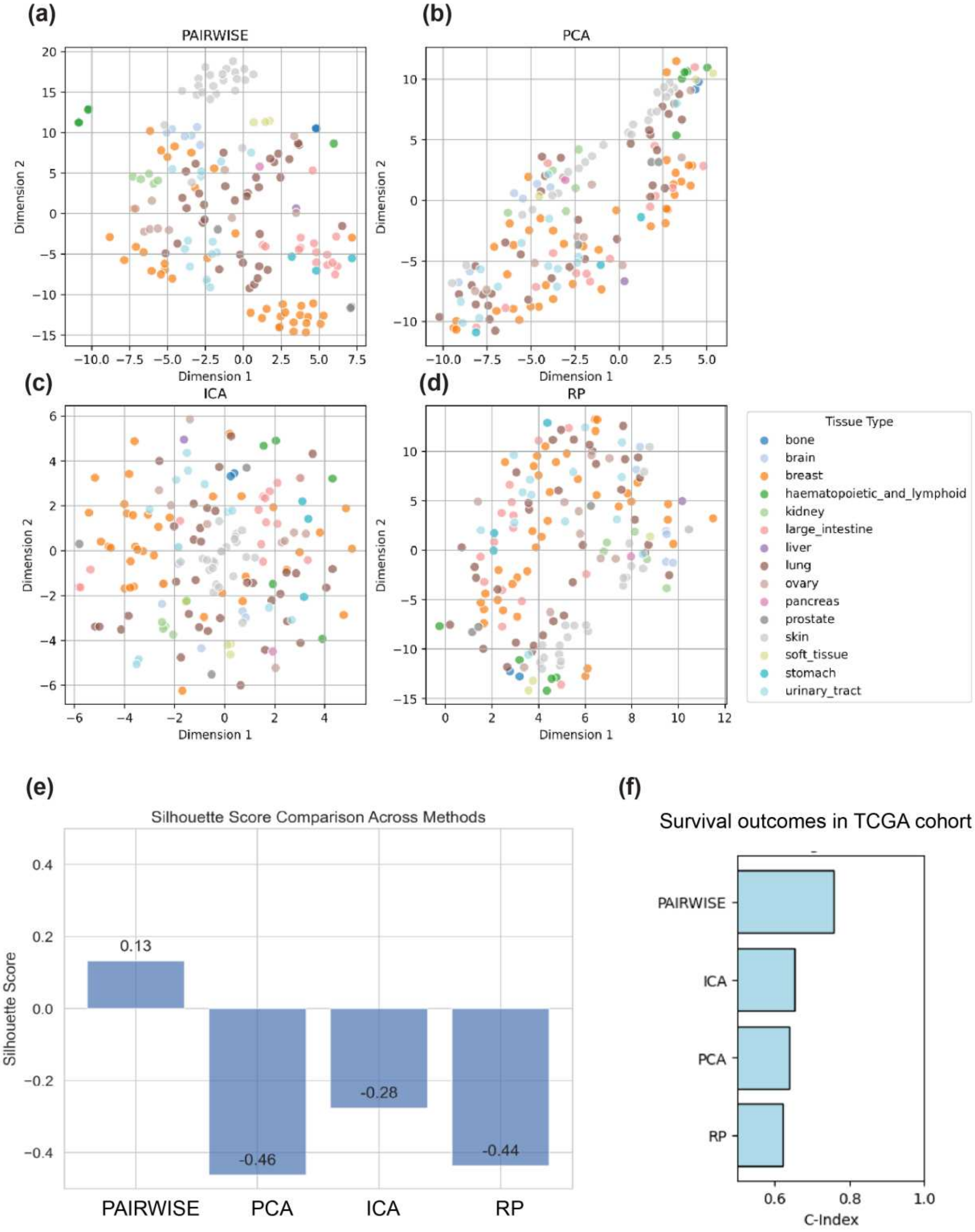

Supplementary Fig. 13. Comparative analysis of gene expression embedding methods for TCGA samples. a-d t-SNE visualization of cancer subtype clustering by different methods: (a) PAIRWISE auto-encoder (AE), (b) Principal Component Analysis (PCA), (c) Independent Component Analysis (ICA), and (d) Random Projections (RP). Each point represents an embedded sample, colored by known cancer subtypes. (e) Silhouette Width comparison across methods, assessing the clustering quality of cancer subtypes. (f) Accuracy of XGBoost models trained with PAIRWISE, PCA, embeddings for predicting survival outcomes.

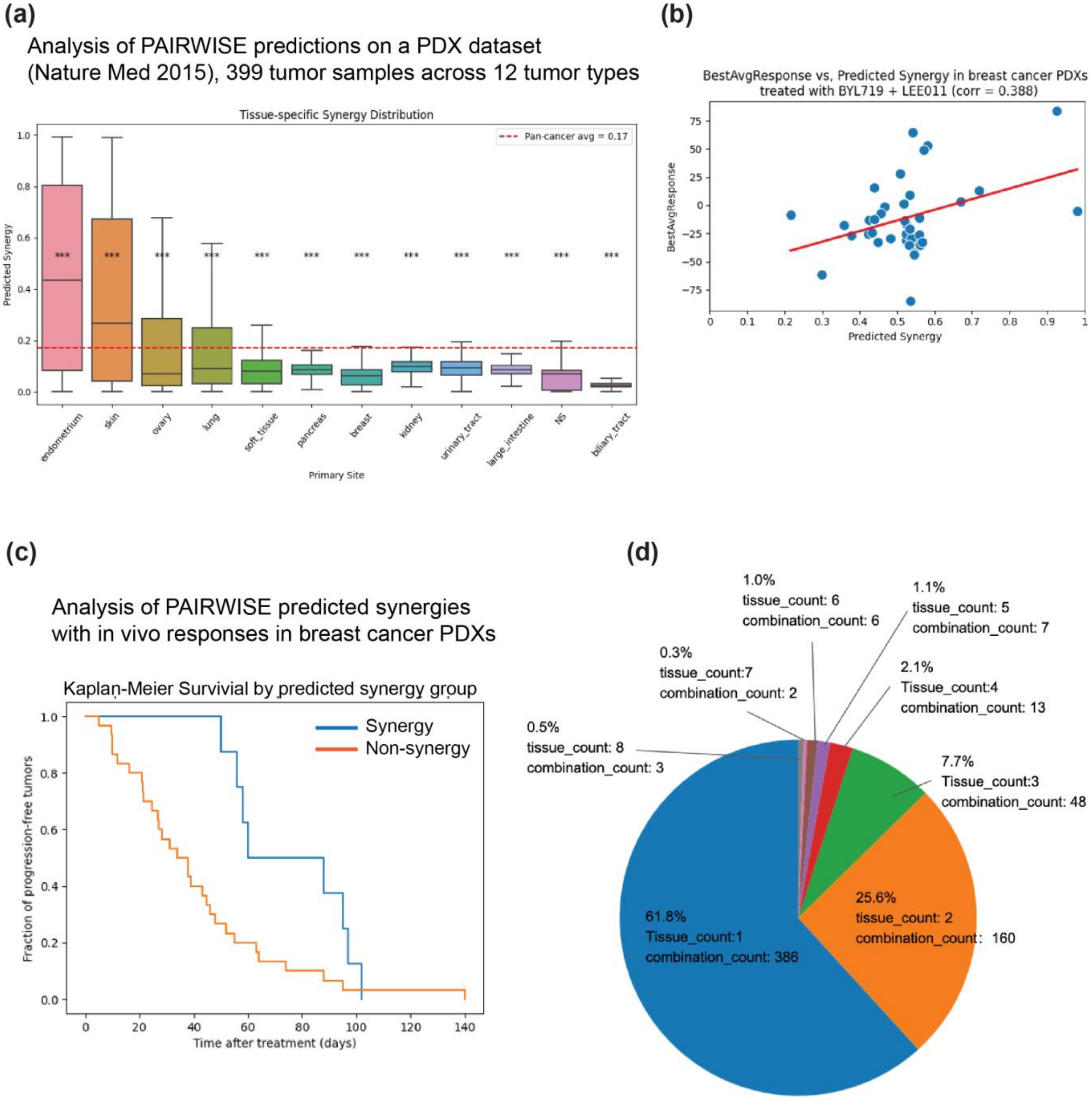

Supplementary Fig. 14. (a) Distribution of predicted synergy scores for drug combinations involving CDK4/6i across 12 types of tumor PDX. In this analysis, we predicted synergy between CDK4/6 inhibition (ribociclib) and other 812 drugs in 399 tumor samples across 12 tumor types. The red line indicates the average predicted synergy across all samples. Paired t-tests compare each tissue against average synergies, with '**' indicating p<0.01 and '***' p<0.001. **(b)** Predicted synergy scores of LEE01 (CDK4/6i) and BYL719 (PI3Ki) drug combination across breast cancer PDX correlate with best average responses of these tumors. The response was determined by comparing tumor volume change at time t to its baseline, indicated as % tumor volume change. **(c)** Survival analysis identifies survival differences between predicted synergistic vs non-synergistic CDK4/6i combinations in PDX samples, log-rank p=0.006. **(d)** Tissue frequency of synergistic drug combinations, showing lineage -specific combination (tissue count=1) unique and multi-cancer predicted synergy (**tissue count>1).**

Supplementary Tables

Study	#experiment	#drug	#cell line	#tissues	Design	Ref
ALMANAC	311,604	103	60	9	4-by-4 or 4-by-6	[9]
MERCK	92,208	38	39	6	5-by-5	[2]
MELANOMA	208,008	108	36	1	3-by-3	[50]
CLOUD	40,160	283	1	1	2-by-2	[51]
ASTRAZENCA-DREAM	11,576	118	85	10	6-by-6	[11]
FLOBAK	9,984	19	8	7	6-by-6 or 10-by-10	[6]
YALE-TNBC	4,576	130	6	1	1-by-5	[52]
YALE-PDAC	3,326	41	4	1	3-by-3	[53]
FORCINA	1,818	1,818	1	1	2-by-2	[54]
GBM	764	31	2	1	6-by-6 or 10-by-10	[55]
YOHE	270	25	3	2	10-by-10	[56]
DECREASE	210	33	13	1	8-by-8	[57]
VISAGE	34	2	34	1	10-by-6	[58]

Supplementary Table. 1 Characteristics of the studied datasets reported in the original paper that are used in this study. The numbers of total experiments, screened drugs, cell lines, tissues and experimental designs (dose-response matrices) are shown below.

Model	Input feature format		Feature encoders		If two drug embeddings are concatenated for inputs in the model
	Cell line	*Drug*	*Cell line*	*Drug*	
Published DL approaches: DeepSynergy	exp	Drug chemical descriptor or Morgan or MACCS fingerprints	DNN	DNN	True
Published DL approaches: MatchMaker	exp	Drug chemical descriptor or Morgan or MACCS fingerprints	DNN	DNN	False
Published DL approaches: Multitask_DNN	exp	Morgan or MACCS fingerprints, DTI from DrugBank V.5.1.10	DNN	DNN	False
Published DL approaches: DeepDDS	exp	SMILES2Graph	MLP	GCN	False
Published DL approaches: Transynergy	exp	Network propagated DTI from DrugBank V.5.1.10 , Morgan fingerprints	Transfor mer	GCN(RWR), Transformer	False
Our DL approaches: **PAIRWISE**	exp	SMILES, DTI from DTC v2.0	Autoenco ders	PFM, DNN	False
PAIRWISE variant model: PAIRWISE-PPI	cell protein association	SMILES, DTI from DTC v2.0	GCN	PFM, DNN	False
PAIRWISE variant model: PAIRWISE-GSVA	GSVA pathway scores	SMILES, DTI from DTC v2.0	DNN	PFM, DNN	False

PAIRWISE variant model: PAIRWISE-SMILES	exp	SMILES, DTI from DTC v2.0	Autoencoders	RDKit library, DNN	False
PAIRWISE variant model: PAIRWISE-Morgan	exp	Morgan fingerprints, DTI from DTC v2.0	Autoencoders	RDKit library, DNN	False
PAIRWISE variant model: PAIRWISE-DrugBank	exp	SMILES, DTI from DrugBank v5.1.10	Autoencoders	PFM, DNN	False
PAIRWISE variant model: PAIRWISE-Propagated DrugBank	exp	SMILES, Network Propagated DTI from DrugBank V.5.1.10	Autoencoders	PFM, DNN	False

Supplementary Table. 2 Benchmarked models for drug combination prediction in our study

Rare Tissues	Training strategy		PAIRWISE	Multitask-DNN
	Train	Test	AUROC	AUROC
Bone tissue	NCATS_ES	NCATS_ES	0.653±0.005	0.598±0.005
	NCATS_DIPG	NCATS_DIPG	0.859±0.012	0.593±0.004
	13 pooled databases	13 pooled databases	0.896±0.009	0.566±0.005
	12 pooled datasets	NCATS_ES	0.678±0.004	0.663±0.003
	ASTRAZENCA-DREAM	NCATS_ES	0.660±0.003	0.540±0.002
Prostate tissue	ONEIL	ONEIL	0.758±0.006	0.650±0.01
	ALMANAC	ALMANAC	0.941±0.004	0.770±0.009
	13 pooled databases	13 pooled databases	0.841±0.001	0.752±0.004
	12 pooled datasets	ONEIL	0.894±0.005	0.637±0.006
	ASTRAZENCA-DREAM	ONEIL	0.667±0.003	0.582±0.004

Supplementary Table. 3 Prediction performance of PAIRWISE and Multitask-DNN on cancer types with limited training data

Model	Drug features	Pearson	Spearman	AUROC	AUPRC	Accuracy
PAIRWISEabCF	Drug target	0.5162 ± 0.0101	0.4864 ± 0.0094	0.8052 ± 0.0058	0.6643 ± 0.0107	0.7758 ± 0.0049
PAIRWISE	Drug target + chemical structure	**0.5624 ± 0.0087**	**0.5306 ± 0.0082**	**0.8550±0.004**	**0.7105 ± 0.005**	**0.7918 ± 0.0047**

Supplementary Table 4. Performance of PAIRWISE without Chemical Fingerprint (PAIRWISEabCF). Pearson and Spearman indicate the correlations between predicted synergy probabilities and experimental synergy scores. AUROC assesses the model's ability to distinguish synergistic versus non-synergistic combinations, while AUPRC evaluates performance in identifying synergistic combinations, particularly for imbalanced datasets. Accuracy represents the proportion of correctly classified drug combinations.

Supplementary Table. 5 Drug screening raw data, see attachment

	Accuracy	95% CI
**Fold 1**	0.725	(0.633, 0.805)
**Fold 2**	0.651	(0.556, 0.739)
**Fold 3**	0.705	(0.611, 0.787)
**Fold 4**	0.732	(0.640, 0.811)
**Fold 5**	0.708	(0.615, 0.789)
**Average**	0.704	(0.611,0.786)

Supplementary Table. 6: Accuracy of DLBCL Bayes predictor in 5-fold cross-validation
